# TRBP2, a Major Component of the RNAi Machinery, Is Subjected to Cell Cycle-Dependent Regulation in Human Cancer Cells of Diverse Tissue Origin

**DOI:** 10.3390/cancers16213701

**Published:** 2024-11-01

**Authors:** Eleni I. Theotoki, Panos Kakoulidis, Athanassios D. Velentzas, Konstantinos-Stylianos Nikolakopoulos, Nikolaos V. Angelis, Ourania E. Tsitsilonis, Ema Anastasiadou, Dimitrios J. Stravopodis

**Affiliations:** 1 Section of Cell Biology and Biophysics, Department of Biology, School of Science, National and Kapodistrian University of Athens (NKUA), 157 01 Athens, Greece; elthk@biol.uoa.gr (E.I.T.); ksnikolakop@biol.uoa.gr (K.-S.N.); 2 Center of Basic Research, Biomedical Research Foundation of the Academy of Athens (BRFAA), 115 27 Athens, Greece; pkakoulidis@di.uoa.gr; 3 Department of Informatics and Telecommunications, School of Science, National and Kapodistrian University of Athens (NKUA), 157 01 Athens, Greece; 4 Section of Animal and Human Physiology, Department of Biology, School of Science, National and Kapodistrian University of Athens (NKUA), 157 01 Athens, Greece; nangelis@biol.uoa.gr (N.V.A.); rtsitsil@biol.uoa.gr (O.E.T.); 5 Department of Health Science, Higher Colleges of Technology (HCT), Academic City Campus, Dubai 17155, United Arab Emirates

**Keywords:** cancer, cell cycle, DICER, miRNA, mitosis, PACT, RISC, RNAi, TRBP2

## Abstract

Background: Transactivation Response Element RNA-binding Protein (TRBP2) is a double-stranded RNA-binding protein widely known for its critical contribution to RNA interference (RNAi), a conserved mechanism of gene-expression regulation mediated through small non-coding RNA moieties (ncRNAs). Nevertheless, TRBP2 has also proved to be involved in other molecular pathways and biological processes, such as cell growth, organism development, spermatogenesis, and stress response. Mutations or aberrant expression of TRBP2 have been previously associated with diverse human pathologies, including Alzheimer’s disease, cardiomyopathy, and cancer, with TRBP2 playing an essential role(s) in proliferation, invasion, and metastasis of tumor cells. Methods: Hence, the present study aims to investigate, via employment of advanced flow cytometry, immunofluorescence, cell transgenesis and bioinformatics technologies, new, still elusive, functions and properties of TRBP2, particularly regarding its cell cycle-specific control during cancer cell division. Results: We have identified a novel, mitosis-dependent regulation of TRBP2 protein expression, as clearly evidenced by the lack of its immunofluorescence-facilitated detection during mitotic phases, in several human cancer cell lines of different tissue origin. Notably, the obtained TRBP2-downregulation patterns seem to derive from molecular mechanisms that act independently of oncogenic activities (e.g., malignancy grade), metastatic capacities (e.g., low versus high), and mutational signatures (e.g., p53^−/−^ or p53^ΔΥ126^) of cancer cells. Conclusions: Taken together, we herein propose that TRBP2 serves as a novel cell cycle-dependent regulator, likely exerting mitosis-suppression functions, and, thus, its mitosis-specific downregulation can hold strong promise to be exploited for the efficient and successful prognosis, diagnosis, and (radio-/chemo-)therapy of diverse human malignancies, in the clinic.

## Simple Summary

Engagement of an advanced immunofluorescence technology demonstrated, for the first time, the cell cycle-dependent control of TRBP2 protein, a major component of the RNAi machinery. TRBP2 expression is lost during mitosis and restored in the cell nucleus upon interphase entry. None of the post-translational modifications, such as ubiquitination or phosphorylation, herein examined proved able to play an essential role in TRBP2 loss from the nucleus of mitotic cells. Different types of cancer cells, with diverse tissue origin, malignancy grade, metastatic potential, and mutational load, are shown to lack TRBP2 nuclear compartmentalization during mitosis, thereby opening new diagnostic and therapeutic windows for human malignancies in the clinic.

## 1. Introduction

Post-transcriptional and translational machinery constitute two of the most important multi-protein assemblies for the time- and tissue-specific control of gene expression in eukaryotic cells. In this context, microRNAs (miRNAs/miRs) are presented as one of the most well-known groups of small non-coding RNAs (ncRNAs) since they have been the main subject of many studies for years. These molecules are endogenous, non-coding, single-stranded RNAs with a size of ~19–25 nucleotides, critically involved in post-transcriptional gene regulation [1,2,3]. In humans, the majority of miRNA sequences are located within intronic regions of coding or non-coding RNA transcripts, whereas some miRNAs are encoded by exonic areas [4].

Biogenesis of miRNA moieties takes place both in the nucleus and the cytoplasm. During canonical biogenesis, each miRNA is transcribed by the RNA Polymerase II (Pol II) as a primary miRNA (pri-miRNA) [5,6,7,8], which is next processed by the RNase III Drosha to generate a precursor miRNA (pre-miRNA) of ~70 nucleotides in length [1,9]. Following Drosha-derived processing, the Ran-GTP-dependent transporter Exportin-5 mediates the translocation of the produced pre-miRNA through the nuclear pore complex (NPC) [10,11,12]. After its export to the cytoplasm, the pre-miRNA is further processed by the RISC (RNA-induced silencing complex) loading complex, composed of DICER, TRBP2 (transactivation response element RNA-binding protein), and PACT (protein activator of the interferon-induced protein kinase) proteins, which cleaves the pre-miRNA hairpins to generate the mature miRNA species [1,13,14]. Once produced, mature miRNA is loaded onto RISC to direct post-transcriptional repression via sequence complementarity to target mRNA(s) [3]. Downregulation of gene expression can be attained through either mRNA cleavage (and degradation) or mRNA translational repression, depending on whether the miRNA presents full or partial complementarity to the target mRNA sequence, respectively [15,16,17,18].

TRBP2 is a double-stranded RNA-binding protein, initially identified due to its ability to bind the HIV-1 TAR RNA and stimulate the expression of the HIV-1 promoter in infected cells [19,20,21]. It is a key component of RISC that, together with PACT, a TRBP2 paralog, can facilitate the DICER-mediated production of miRNA species [22]. TRBP2 has been previously shown to stabilize DICER and enhance DICER-catalyzed miRNA-processing kinetics [23,24,25], as well as DICER’s ability to recognize pre-miRNAs among other RNA species [25,26,27,28]. In addition, TRBP2 is required for the loading of miRNA duplex onto AGO2 during RISC assembly, while it can also, indirectly, affect the selection of guide strand during mRISC formation due to a shift of cleavage sites [26,27,29,30,31].

Two major isoforms of the protein, TRBP1 and TRBP2, encoded by the *TARBP2* gene located on chromosome 12, coexist in human cells [32,33,34,35]. These two proteins do not seem to contain major differences in their functionalities, although TRBP2 is presented as slightly more active than TRBP1 in human cells [32]. The three TRBP(1/2) main domains, ordered from NH_2_- to COOH-terminus, are named (a) dsRBD1 and (b) dsRBD2, located in between 31–96 and 160–226 amino acid residues (aa), respectively, and (c) C4, positioned in between 298–366 aa [19,27,36]. The two dsRBDs can bind dsRNAs, with dsRBD2 carrying stronger dsRNA-binding activity than dsRBD1 due to the presence of a KR-helix motif, a 15 aa peptide sequence within dsRBD2, which can bind dsRNA by itself [36,37,38,39]. C4, also called half dsRBD or dsRBD type B, has structural homologies with dsRBDs, but it does not bind RNA. In contrast, the C4 domain mediates Protein–Protein Interactions (PPIs) in an RNA-independent manner [40,41,42]. Importantly, C4 has proved responsible for the TRBP2-DICER and TRBP2-PACT interactions, while a larger region (237–366 aa) encompassing C4, known as the “Medipal” region, has been previously identified for the TRBP2-MERLIN interaction [32,42,43,44].

The two TRBP1 and TRBP2 proteins are almost identical to each other, and they only differ in the presence of a 21 aa sequence, located specifically at the NH_2_-terminal end of TRBP2, a consequence of two adjacent-promoter activities that control the alternative synthesis of *TARBP1* (*TARBP2-202*) and *TARBP2* (*TARBP2-201*) mRNA transcripts, with *TARBP2* first exon encoding for the additional 21 aa peptide (Supplementary Figure S1A,B) [19,21,35,43]. Nevertheless, it is the extremely high identities in their primary-, secondary-, tertiary-, and motif-based structures (Supplementary Figure S1B–E) that enabled us to investigate only the TRBP2 protein by the reasoning of its (TRBP2) strong functional similarities to the TRBP1 respective ones.

Remarkably, besides its fundamental contribution to the operation of RNAi machinery, TRBP2 has been recently implicated in the critical orchestration of miRNA/RNAi-independent processes, including, among others, (a) osteosarcoma-cell proliferation and invasion [45], (b) hepatocellular-carcinoma progression [46], and (c) gastric cancer growth and metastasis [47], thus rendering TRBP2 a major determinant of the malignant phenotype. It must be the proficiency of TRBP2 to play essential roles in the control of cell survival [48], apoptotic death [49], and mitosis [49,50] that obligates the mechanistic illumination of TRBP2 functionality during cancer-cell division, growth, migration, and resistance to therapy.

Given the limited knowledge regarding the regulatory coupling of TRBP2 with mitosis-apparatus activities, the present study aims to investigate the hitherto unknown and likely miRNA/RNAi-independent functions and properties of the TRBP2 protein, particularly regarding its cell cycle-dependent regulation during cancer-cell division. Our novel findings of the mitosis-specific TRBP2, but not PACT, downregulation in human cancer cells of diverse tissue origin provide strong insights for the essential involvement of TRBP2 in the onset and progression of human malignancies and highlight the TRBP2 protein as a new and promising biomarker for the efficient and successful prognosis, diagnosis, and (radio-/chemo-)therapy of the disease.

## 2. Materials and Methods

### 2.1. Cell Lines—Culture Conditions

NTHY-ori 3-1, TPC-1, and ARO cell lines were cultured in RPMI 1640 growth medium (61870-010, Gibco, Thermo Fisher Scientific, Waltham, MA, USA), while RT112, T24, TCCSUP, HCT116-p53^+/+^, HCT116-p53^−/−^, WM115, WM266-4, LX-2, and HepG2 cell lines were cultured in DMEM (41966-029, Gibco, Thermo Fisher Scientific, Waltham, MA, USA) in standard conditions (+37 °C and 5% CO_2_). The medium was supplemented with 10% FBS (16000044, Gibco Thermo Fisher Scientific, Waltham, MA, USA), 1% penicillin/streptomycin (10378016, Gibco, Thermo Fisher Scientific, Waltham, MA, USA), and 1% L-glutamine (BEBP17-605E, Lonza, Verviers, Belgium).

### 2.2. Immunofluorescence

Cells were cultured on 10 mm round coverslips and subsequently fixed in 4% paraformaldehyde (P6148, Sigma-Aldrich, St. Louis, MO, USA) solution for 10 min at room temperature. They were membrane-permeabilized by treatment with 0.1% Triton-X 100 (Sigma-Aldrich, St. Louis, MO, USA), followed by blocking with 5% BSA (fraction V) for 1 h. Next, cells were exposed to the anti-TRBP (ab180947, Abcam, Cambridge, UK), anti-PACT (13490, Cell Signaling Technology, Danvers, MA, USA), anti-ATF4 (ab245, GenScript, Piscataway, NJ, USA), and anti-α-Tubulin (3873, Cell Signaling Technology, Danvers, MA, USA) primary antibodies overnight at +4 °C. Finally, they were incubated with the Goat anti-Mouse IgG (H + L), Alexa Fluor™ 568 (A-11004, Invitrogen, Thermo Fisher Scientific, Waltham, MA, USA), and Donkey anti-Rabbit IgG (H + L), Alexa Fluor™ 488 (A-11004, Invitrogen, Thermo Fisher Scientific, Waltham, MA, USA) secondary antibodies for 1 h at room temperature. Vectashield^®^ Mounting Medium with DAPI (Vector Laboratories Inc., Newark, CA, USA) was used to visualize the nuclei at the 405 nm excitation wavelength.

### 2.3. Transient Transfection

LX-2 cells were transiently transfected with the plasmid pcDNA-TRBP (15666, Addgene, Watertown, MA, USA) using Lipofectamine 2000 (11668027, Invitrogen, Thermo Fisher Scientific, Waltham, MA, USA), according to the manufacturer’s instructions. Cells were collected 48 h post-transfection and processed for further assays.

### 2.4. Pharmacological Interventions—Inhibitors

LX-2 cells were treated with 1 µM TAK-981 (32741, Cayman Chemicals, Ann Arbor, MI, USA), 1 µM MLN-4924 (85923, Cell Signaling Technology, Danvers, MA, USA), 1 µM Bortezomib (2204, Cell Signaling Technology, Danvers, MA, USA), 100 µM U0126 (S1102, Selleckchem, Cologne, Germany), 50 µM SP600125 (S1460, Selleckchem, Cologne, Germany), 100 µM SB203580 (S1076, Selleckchem, Cologne, Germany), and 25 µM MK-2206 (S1078, Selleckchem, Cologne, Germany) for 24 h. Additionally, cells were treated with 0.4 µg/µL Demecolcine (D7385, Sigma-Aldrich, St. Louis, MO, USA) for 6 h. Following treatment, the cells were collected for further assays. DMSO (A3672, Sigma-Aldrich, St. Louis, MO, USA) was used as control (to exclude “solvent effect”).

### 2.5. Cell-Cycle Analysis—Flow Cytometry (FACS)

The variation in the expression levels of TRBP2 protein at different phases of the cell cycle was evaluated and quantified after anti-TRBP/IgG-FITC and Propidium Iodide (PI) staining. Briefly, human urothelial bladder cancer cells (T24) were cultured for 24 h in the absence or presence of 5 µg/mL Brefeldin A (BFA; Applichem GmbH, Darmstadt, Germany). After harvesting, cells were washed, fixed in 70% ethanol for 30 min at −20 °C, and, next, incubated with the primary antibody anti-TRBP (3 µg/mL) for 30 min at +4 °C. After washing, cells were incubated for another 30 min at +4 °C in the dark with the anti-Rabbit IgG, FITC-labeled secondary antibody (3 µg/mL). Ribonuclease A (RNase A; 100 µg/mL; Merck, Darmstadt, Germany) was added to ensure PI-staining only of DNA for 30 min at +37 °C. Cells were further stained with PI (25 µg/mL) (Biolegend, San Diego, CA, USA) for 15 min at room temperature in the dark. Analysis was performed on a BD FACSCelesta™ Flow Cytometer (BD Biosciences, Heidelberg, Germany) using the FACSDiva™ version 9.0 software (BD Biosciences, Heidelberg, Germany).

### 2.6. Quantitative PCR (qPCR)

Total RNA from transiently transfected LX-2 cells was extracted using peqGOLD TriFast (30–2010, Avantor/VWR Life Science, Radnor, PA, USA), and cDNA was, next, synthesized using the PrimeScript RT Reagent Kit with gDNA Eraser (RR047A, TaKaRa Bio, Kusatsu, Japan), according to manufacturers’ recommendations. cDNA was amplified in duplicates on a Roche Light Cycler 96 System. Relative gene expression was calculated using the 2^−ΔCt^ method. Expression of the *TARBP2* gene was normalized by the reference gene GAPDH.

### 2.7. Mitotic-Cell Rate Quantification

Transiently transfected and inhibitor-treated LX-2 cells were collected and, subsequently, observed in a Confocal Laser Scanning Microscope (CLSM) after their exposure to the Vectashield^®^ Mounting Medium with DAPI (Vector Laboratories Inc., Newark, CA, USA), followed by random-field photography and cell counting. Each experiment was repeated three times, and approximately 300 cells per experiment were counted.

### 2.8. Protein Molecular Modeling

All structures were pre-processed for docking, engaging the Protein Preparation Wizard of the Schrödinger Maestro suite [51]. Experimental structures were retrieved from PDB REDO [52]. Proteins lacking experimental structural data (e.g., TRBP2 or PACT) were retrieved from AlphaFoldDB [53]. Predicted structures were further refined with locPREFMD [54]. The TRBP1 structure is derived from the TRBP2 structure by truncating the first loop. Machaon’s API method [55] was employed to extract the secondary structures and conduct pairwise alignments. Alignments were visualized via the Biotite Python package, with a coloring matrix suited for 2D alignments. Each structure was docked independently of the others, using the DRBM1 (dsRBD1), DRBM2 (dsRBD2), and DRBM3 (C4) domains of the TRBP2 protein. Docking tests were conducted with the ClusPro web server [56] and assessed with the Prodigy web server [57] to predict the dissociation constants of each complex. In the case of Tubulin A1A, docking was constrained to the exposed residues of its bound form in the cytoskeleton network with other Tubulin family members. PDB 6WSL includes monomers of Tubulin A1A and Tubulin B3 in a complex, and PDBsum [58] was applied to determine the interacting residues of the Tubulin A1A monomers in the complex. The residue positions were combined, discarding the duplicate positions, and were given as repulsion areas for the constrained docking. Two docking operations were conducted for each structure-domain pair, alternating the rigid molecule/flexible ligand roles, as supported by ClusPro. A total of 68 docking operations were carried out and evaluated, in total: [11 proteins × 3 domains × 2 alternative docking roles] and [1 domain × 2 alternative docking roles]. Dockings were selected based on their lowest predicted dissociation constants (K_d_). A K_d_ heatmap plot was produced using the Seaborn version 0.12 Python package. The lowest values are presented with a darker tone, referring to stronger association dynamics.

### 2.9. Statistical Analysis

All statistical analyses were conducted using the IBM SPSS version 26, and the obtained results were presented as mean ± SSD (Sample Standard Deviation). Significance was evaluated using Student’s *t*-test.

## 3. Results

### 3.1. TRBP2-Protein Levels Increase During Cell-Cycle Progression: A G2/M-Enriched Accumulation

**Figure 1:**
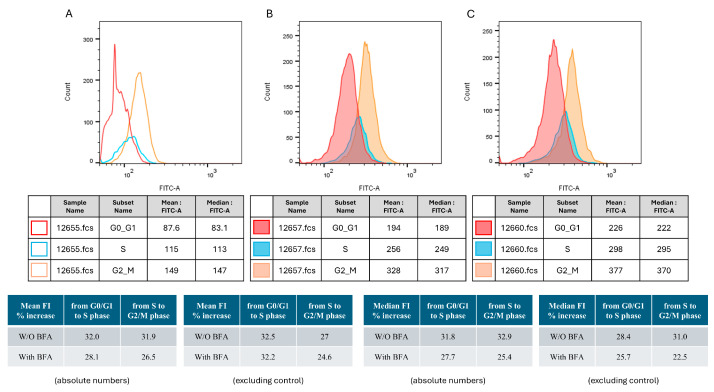
Variation in TRBP2 protein levels in T24 cells at the cell cycle phases. Histograms showing fluorescence distribution of the FITC-conjugated anti-TRBP antibody in each phase of the cell cycle (**A**) under control conditions (PI staining), (**B**) after antibody and PI staining, and (**C**) after Brefeldin A pre-treatment and antibody and PI staining. Values in the tables show the mean fluorescence intensity (MFI) (mean FITC-A) from one representative experiment out of three performed.

To assess TRBP2 levels during the different phases of the cell cycle, we performed flow cytometry analysis of T24 human urothelial bladder-cancer cells (malignancy grade III; p53^ΔY126^), stained with anti-TRBP antibody and PI. The protein-secretion inhibitor Brefeldin A (BFA) was used for comparison (Figure 1). To identify the FITC-positive cells in each of the interphase stages, the gating strategy depicted in Supplementary Figure S2 was used. The mean fluorescence intensity (MFI) of T24 cells showed a gradual increase from G0/G1, through S, to G2/M (194 vs. 256 vs. 328, respectively) (Figure 1B). Pre-treatment of T24 cells with BFA did not significantly alter MFI values, which similarly increased during cell-cycle progression (226, 298, and 377, for G0/G1, S, and G2/M, respectively) (Figure 1C). Upon exclusion of the background signal (Figure 1A), the MFI (%) increase from G0/G1 to S was 32.5% and from S to G2/M was 27% for T24 cells being cultured in the absence of BFA. Interestingly, the respective (% MFI) values (G0/G1-S and S-G2/M) for the pre-treated with BFA (T24) cells were measured as 32.2% and 24.6%, respectively, thereby indicating that TRBP2 is not transported extra-cellularly in urothelial-carcinoma settings.

### 3.2. Interphase-Specific Nuclear Accumulation of the TRBP Protein in Human Thyroid Cells

**Figure 2:**
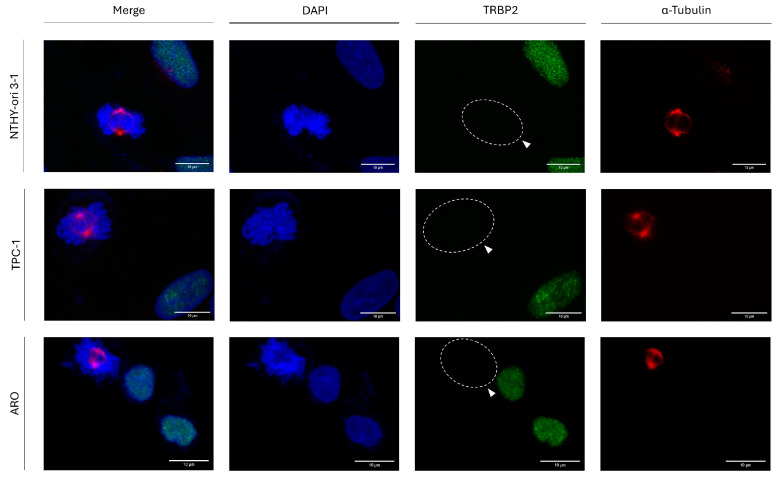
TRBP2 expression profiles at the interphase and mitosis of thyroid cells. Immunofluorescence images of NTHY-ori 3-1, TPC-1, and ARO cells, investigating TRBP2 expression and distribution patterns in both interphase and mitotic cells. In cells undergoing mitosis, a lack of TRBP2-protein immunodetection is observed (white arrows). Green color: TRBP2; Red color: α-Tubulin; Blue color: Nucleus (DAPI). Scale bars: 10 µm.

Since TRBP2 presents the highest level of protein accumulation at the G2/M phase of the T24 cell cycle (Figure 1), we next examined its (TRBP2) immunofluorescence-facilitated detection during interphase and mitosis, attempting to distinguish TRBP2′s accumulation profiles in between G2 and M cell-cycle phases. Hence, we thoroughly investigated its protein expression, distribution, and accumulation during both interphase and mitosis in three different human thyroid cell lines (Figure 2). Surprisingly, a complete absence of TRBP2 immunodetection during cell division and specifically mitosis (determined by α-Tubulin/microtubules immunostaining and mitotic spindle formation) was observed in all the examined cells of (a) the normal thyroid cell line NTHY-ori 3-1 (human thyroid follicular epithelial cells) and (b) the thyroid cancer cell lines TPC-1 (human papillary thyroid carcinoma) and ARO (human thyroid anaplastic carcinoma) herein used. Strikingly, in contrast to mitotic cells, interphase cells were characterized by strong TRBP2 nuclear signals (Figure 2).

These results indicate a novel regulatory role of TRBP2 in cell cycle progression. It is the remarkably reduced expression of the protein (TRBP2) that seems harmful for the successful implementation and completion of thyroid cell mitosis, regardless of the immortalized (NTHY-ori 3-1) or oncogenic (TPC-1 and ARO) cellular backgrounds.

### 3.3. Mitosis-Dependent Downregulation of TRBP2 Expression in Human Urothelial Bladder Cancer Cells

**Figure 3:**
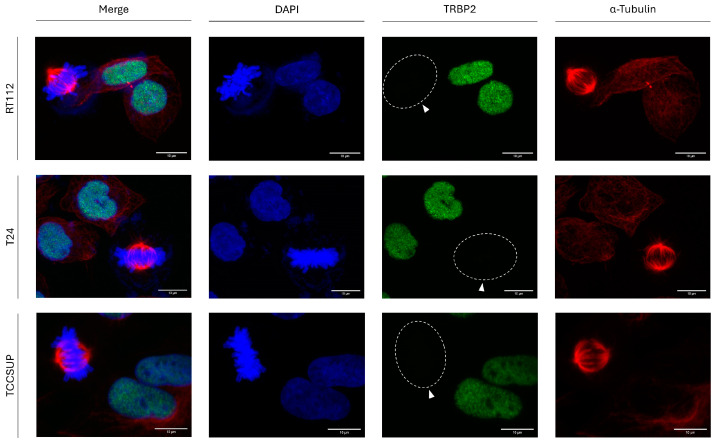
Downregulation of TRBP2 protein expression in urothelial bladder cancer cells during mitosis. Immunofluorescence images of RT112, T24, and TCCSUP cells, searching for TRBP2 expression and distribution profiles in both interphase and mitotic cells. In dividing cells, the absence of TRBP2 immunostaining is detected at the mitosis stage (white arrows). Green color: TRBP2; Red color: α-Tubulin; Blue color: Nucleus (DAPI). Scale bars: 10 µm.

To investigate whether mitosis-dependent downregulation of TRBP2 is specific for thyroid cancer cells, we next examined its (TRBP2) protein expression profiles in the three human urothelial bladder-cancer cell lines (a) RT112 (malignancy grade I/II), (b) T24 (malignancy grade III), and (c) TCCSUP (malignancy grade IV). Most importantly, it proved that TRBP2 immunodetection follows the same pattern, just like in thyroid cells, for all the three different bladder cancer cell lines herein studied, with TRBP2 protein expression being completely missing from mitotic cells but readily detected in interphase bladder-cancer cell nuclei for all analyzed cells (Figure 3). Our findings not only demonstrate the universality of this novel phenomenon but also dictate the functionality of a common molecular mechanism that can act independently of the malignancy grade and tumor tissue origin, thus strongly supporting the essential contribution of TRBP2 as a negative regulator to successful mitosis implementation in bladder carcinoma environments.

### 3.4. p53 Protein Activity Is Not Required for TRBP2 Downregulation During Mitosis in Human Colon Cancer Cells

**Figure 4:**
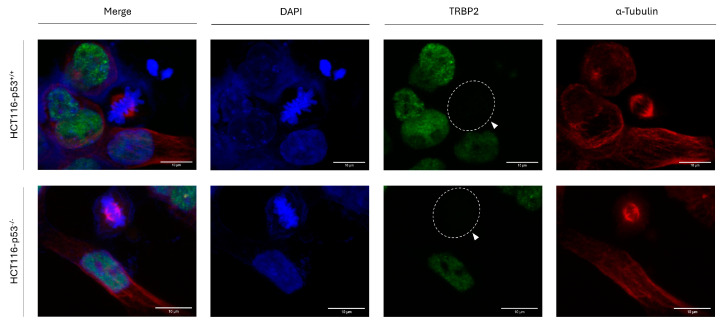
p53-independent downregulation of TRBP2 protein in colon carcinoma-dividing cells. Immunofluorescence images of HCT116 colon cancer cells, seeking for TRBP2 expression and distribution profiles either in the presence (HCT116-p53^+/+^) or in the absence (HCT116-p53^−/−^) of the wild-type p53 protein form. In both cases, TRBP2 elimination from dividing cells during mitosis is identified (white arrows). Green color: TRBP2; Red color: α-Tubulin; Blue color: Nucleus (DAPI). Scale bars: 10 µm.

Considering that, in contrast to RT112, T24 cells carry a loss-of-function mutation in the *TP53*-gene locus (p53^ΔΥ126^) [59], we next reasoned that the tumor suppressor protein p53 may not get involved in the TRBP2 regulation during cell division. To further examine our argument in a setting that, besides a *TP53*-detrimental deletion, does not contain additional genetic lesions, we next used the human colon cancer cell line HCT116-p53^−/−^ that lacks *TP53*-gene activity to check TRBP2-expression profile during mitosis in the absence of p53 protein, with the parental HCT116-p53^+/+^ counterpart colon cancer cells that express the wild-type p53-protein form being used as suitable control. Both HCT116-p53^−/−^ and HCT116-p53^+/+^ cell types were characterized by the disappearance of TRBP2 immunofluorescence-mediated (TRBP2-negative) detection exclusively in mitotic cells, with all interphase cells exhibiting strong nuclear (TRBP2-positive) signals (Figure 4). Our results clearly unveil a p53-independent mechanism of TRBP2 downregulation during mitosis, which seems to act for bladder and colon carcinomas in highly similar fashions, regardless of the tumor tissue origins.

### 3.5. Metastasis-Independent Control of TRBP2 Elimination During Human Melanoma Cell Division

**Figure 5:**
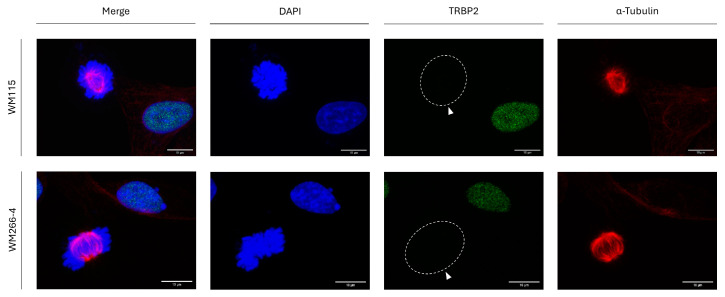
Metastasis-independent TRBP2 downregulation during melanoma cell division. Immunofluorescence images of pre-metastatic WM115 and metastatic WM266-4 melanoma cells, exploring TRBP2 expression and compartmentalization patterns in both interphase and mitotic cells. Lack of TRBP2 immunodetection profiles in mitotic cells of both cell lines is observed (white arrows). Green color: TRBP2; Red color: α-Tubulin; Blue color: Nucleus (DAPI). Scale bars: 10 µm.

Given that RT112 and T24 cells also differ in their metastatic features, with T24 presenting tumor xenograft-emanated strong metastatic activities in SCID (severe combined immunodeficient) mice (Material intended for publication), metastasis-controlling programs do not seem to be critically involved in TRBP2 elimination during mitosis. To further expand and strengthen our rationale, we next analyzed the TRBP2 protein-expression profiles in pre-metastatic (WM115) and metastatic (WM266-4) human melanoma cells, both having been derived from the same patient (thus, excluding a plethora of genetic variations, polymorphisms, and metastasis-irrelevant mutations). Since WM115 and WM266-4 human melanoma cell lines are both characterized by the lack of TRBP2 detection in all mitotic cells and by the simultaneous TRBP2 presence in all interphase-cell nuclei examined (Figure 5), metastasis-dependent mechanisms must play non-essential or dispensable/redundant, roles in mitosis-specific TRBP2 elimination in human melanoma (and urothelial bladder carcinoma; Figure 3) settings.

### 3.6. Oncogenesis-Independent Loss of TRBP2 Immunodetection in Human Hepatic Cells Undergoing Mitosis

**Figure 6:**
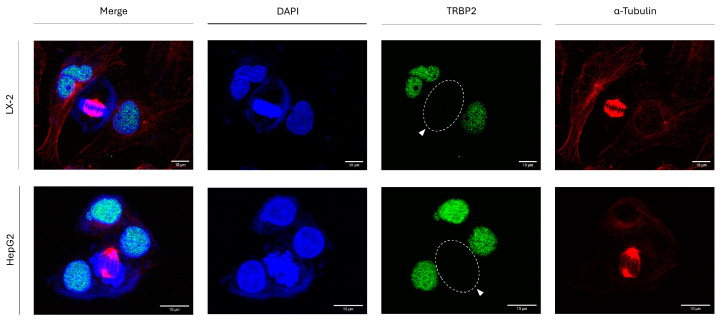
Oncogenesis-independent control of TRBP2 expression in hepatic cells undergoing mitosis. Immunofluorescence images of LX-2 and HepG2 cells directly reflect TRBP2 expression and distribution patterns in human hepatic cells. In dividing cells, loss of TRBP2 protein is detected during mitosis (white arrows). Green color: TRBP2; Red color: α-Tubulin; Blue color: Nucleus (DAPI). Scale bars: 10 µm.

To examine the oncogenicity network independence of TRBP2-protein loss in a cellular system other than thyroid cell mitosis (Figure 2), we next investigated the TRBP2-expression patterns in human hepatic stellate (LX-2) and human hepatocellular carcinoma (HepG2) cells during cell division. Similarly to human thyroid cells (Figure 2), both LX-2 and HepG2 human hepatic cells are presented with simultaneous profiles of mitosis-specific TRBP2 loss and interphase-exclusive TRBP2 accumulation in the nuclear compartment of all analyzed cells (Figure 6). Taken together, it seems that both thyroid and hepatic cells engage indistinguishable mechanisms (a) to strongly retain TRBP2 in the nucleus during interphase and (b) to specifically eliminate TRBP2 in mitosis-subjected cells, following oncogenesis-independent patterns. It is neither the oncogenic mutational signature(s) nor the tumor tissue origin(s) that can control the universality of the TRBP2 loss phenotype in human cells undergoing mitosis.

### 3.7. Mitotic Phase-Dependent Control of TRBP2 Downregulation in LX-2 Hepatic Cells

**Figure 7:**
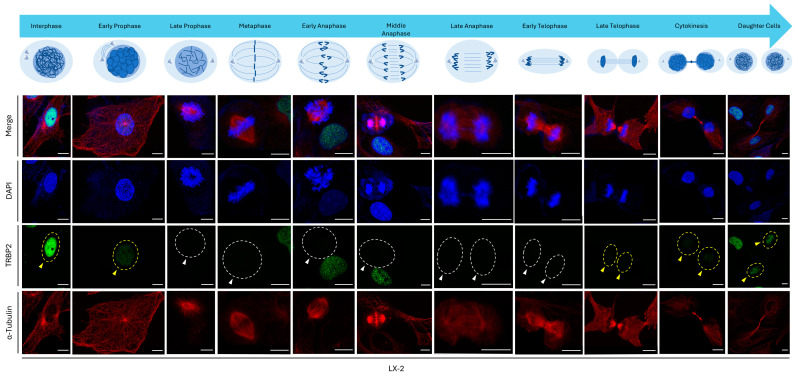
Cell cycle-dependent regulation of TRBP2-protein expression and compartmentalization in hepatic cells. Immunofluorescence images of LX-2 hepatic cells unveil TRBP2 expression and distribution patterns at different stages of mitosis. The presence (yellow arrows) or the absence (white arrows) of the TRBP2 protein immunodetection can be distinguished at each stage of the cell division process. Green color: TRBP2; Red color: α-Tubulin; Blue color: Nucleus (DAPI). Scale bars: 10 µm.

Given the major alteration (loss) of immunofluorescence-facilitated TRBP2 expression imaging during mitosis, we further examined TRBP2 immunodetection profiles at each individual stage of the mitotic process, namely, from interphase and early prophase to late telophase, cytokinesis, and daughter cell stages. Remarkably, LX-2 hepatic cells showed a strong TRBP2 signal in the interphase nucleus, which was significantly reduced at the early prophase stage and completely lost at late prophase (Figure 7). TRBP2 nuclear staining was eliminated from late-prophase to early-telophase (including metaphase, early anaphase, middle anaphase, and late anaphase) stages, while its (TRBP2) signal was gradually captured again from late telophase to cytokinesis and daughter cell stages that marked the completion of mitosis (Figure 7). Of note, the TRBP2 nuclear signal was fully recovered during the formation of the two daughter cells, which defined the completion of LX-2 hepatic cell division (Figure 7). This tight control for the TRBP2 loss during mitosis could be recognized in all the examined LX-2 hepatic cells, rendering TRBP2 a novel, negative regulator for mitotic apparatus and cell proliferation and growth machinery.

### 3.8. Hepatic and Colon Cancer Cells Express PACT Protein During Both Interphase and Mitosis Stages

**Figure 8:**
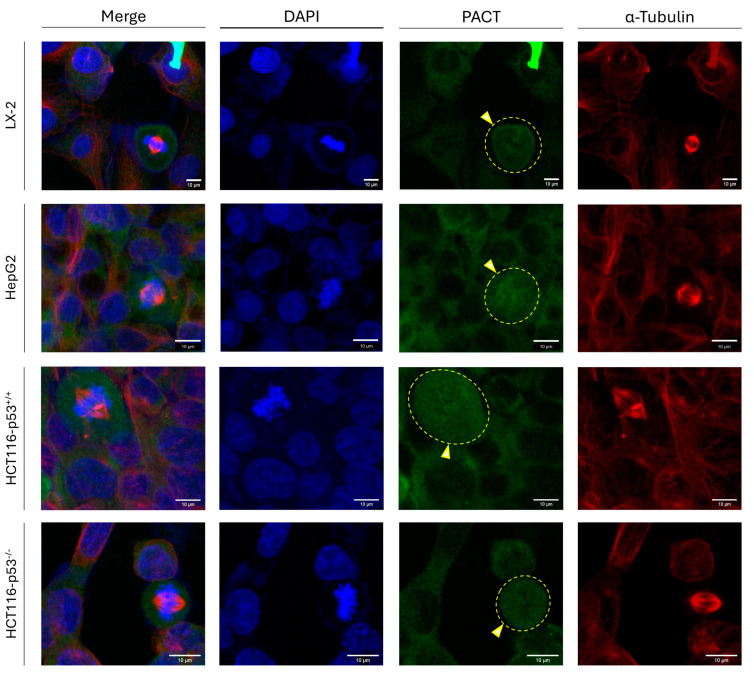
PACT protein expression profiling at the interphase and mitosis of dividing cells. Immunofluorescence images of LX-2, HepG2, and HCT116 cells, investigating PACT expression and distribution in both interphase and mitotic cells. PACT-immunodetection pattern is found diffused in the cytoplasm, whereas it maintains an intense signal during mitosis (yellow arrows) in all the examined cell lines, regardless of the malignant phenotype (LX-2 versus HepG2) or the absence of the wild-type p53 protein (HCT116-p53^+/+^ versus HCT116-p53^−/−^). Green color: PACT; Red color: α-Tubulin; Blue color: Nucleus (DAPI). Scale bars: 10 µm.

Since PACT (a) constitutes a TRBP2 paralog, (b) presents remarkable similarities in its primary, secondary (2D), and tertiary (3D) structures to the TRBP2 respective ones (including in silico motifs) (Supplementary Figure S1A–E), (c) interacts with TRBP2, (d) belongs to the RISC-loading complex, and (e) facilitates the DICER-mediated production of miRNAs [22], we next investigated the PACT-specific expression profiles in cells undergoing mitosis, or residing at the interphase stage of their cell cycle. Strikingly, at interphase, hepatic (LX-2 and HepG2) and colon cancer (HCT116-p53^+/+^ and HCT116-p53^−/−^) (human) cell lines were characterized by a mainly cytoplasmic pattern (absence of nuclear staining) of PACT distribution, while, at mitosis, PACT maintained its strong, albeit diffuse, immunodetection signal that was being dispersed throughout all the mitotic cells examined (Figure 8). The obtained phenotypes remained unaffected either by the oncogenic status (LX-2 versus HepG2) or by the p53-mutational signature (HCT116-p53^+/+^ versus HCT116-p53^−/−^) for both hepatic and colon cancer cells herein used (Figure 8).

Taken together, it seems that, in contrast to TRBP2, PACT protein retains its immunofluorescence-derived detection pattern (strong and dispersed expression throughout the cell) unharmed during mitosis, thus indicating the ability of PACT to function independently of TRBP2 at the mitotic stage. Given that TRBP2, but not PACT, is exclusively compartmentalized in the nucleus at interphase and is lost at mitosis, whereas PACT seems to be strongly distributed both in the cytoplasm and the nucleus at mitosis (and not in the nucleus at interphase), PACT and TRBP2 may operate reversely to each other, with TRBP2 inhibiting and PACT promoting, RNAi-independent, mitotic functions (e.g., PKR (kinase) activities [49,50]), in accordance with their opposite, respective actions in innate immunity and anti-viral signaling, recently reported [60].

### 3.9. Overexpression of TRBP2 Protein Cannot Rescue Its Mitosis-Specific Loss in LX-2 Dividing Cells

**Figure 9:**
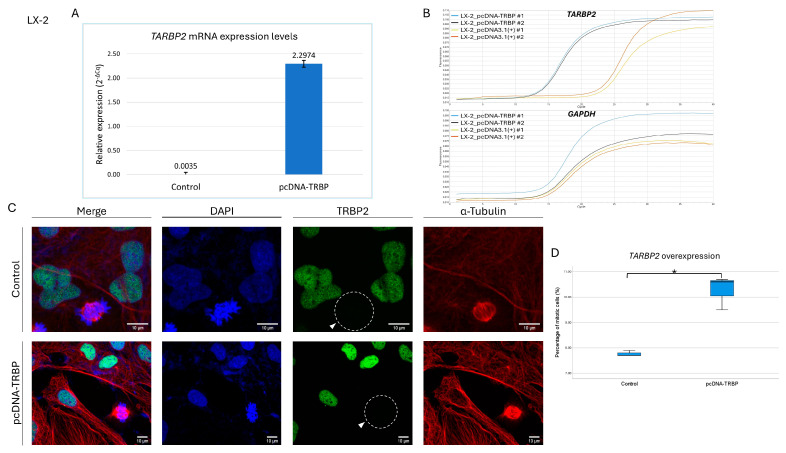
Overexpression of TRBP2 protein in LX-2 hepatic cells cannot rescue its mitosis-specific loss. (**A**) Histogram presenting the relative expression levels of the *TARBP2* gene in *TARBP2* transiently transfected LX-2 cells (pcDNA-TRBP), as compared to control cells (pcDNA3.1(+)). (**B**) Amplification curves (in duplicates) showing the increase in fluorescence over the qPCR cycles for *TARBP2* and *GAPDH* (reference gene) mRNAs in *TARBP2*-overexpressing and control LX-2 cells. (**C**) Immunofluorescence images of LX-2 cells transiently overexpressing the TRBP2 protein, searching for TRBP2 expression in both interphase and mitotic cells, 48 h post-transfection. TRBP2 has been lost during mitosis in both overexpressing (pcDNA-TRBP) and control (pcDNA3.1(+)) cells (white arrows). Green color: TRBP2; Red color: α-Tubulin; Blue color: Nucleus (DAPI). Scale bars: 10 µm. (**D**) Box-plot presenting the percentage of mitotic-cell numbers (%) in *TARBP2*-overexpressing LX-2 cells (pcDNA-TRBP) as compared to control (pcDNA3.1(+)) cells (*: *p* < 0.05).

Next, we considered if the increased expression levels of TRBP2 protein may have an impact on its loss from the nuclear compartment during mitosis. Therefore, we transiently transfected LX-2 cells with the pcDNA-TRBP construct (using Lipofectamine 2000) to overexpress *TARBP2*, with LX-2 treated only with Lipofectamine and the empty vector being used as a control. Additionally, 48 h post-transfection, cells were carefully collected and immediately processed for immunofluorescence imaging. Results showed that overexpression (~650×) of *TARBP2* (Figure 9A,B) did not alter its expression profile in dividing cells, as the TRBP2 protein was missing from both control and *TARBP2*-overexpressing cells undergoing mitosis (Figure 9C). Nevertheless, the overexpression of *TARBP2* proved to critically affect the proliferation rate, as it significantly increased the number of dividing cells (Figure 9D). Intriguingly, the mean percentage of mitotic cell number in the case of *TARBP2* overexpression was ~10.3%, compared to ~7.7% of the control, an increase that dictates the propensity of upregulated TRBP2 to cause late-interphase/early-mitosis enhanced activities. Altogether, we suggest that TRBP2 upregulation may promote the entry to early mitosis, whereas TRBP2 downregulation (and loss) is likely required for the progression from early to late mitosis and, finally, for the successful entry to the early-interphase stage of the next cell cycle.

### 3.10. Dispensable Roles of SUMOylation-, NEDDylation-, and Proteasome-Dependent Pathways in the Mitosis-Specific Loss of TRBP2-Immunodetection Profile in LX-2 Hepatic Cells

**Figure 10:**
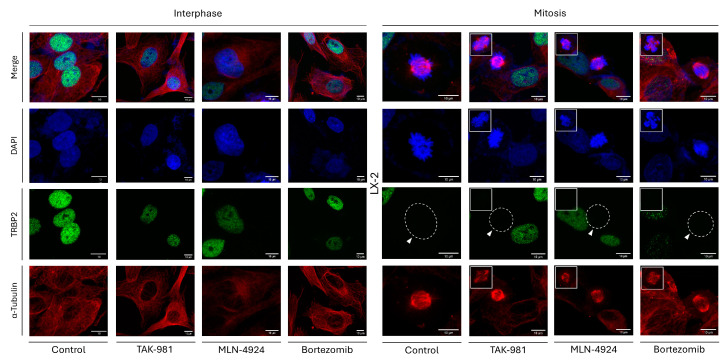
Chemical inhibition of SUMOylation-, NEDDylation-, and Proteasome-dependent types of machinery cannot alter the TRBP2 immunostaining patterns in LX-2 hepatic cells. Immunofluorescence images of LX-2 cells, seeking TRBP2 expression in interphase (**left panels**) and mitotic (**right panels**) cells, 24 h after the administration of 1 µM TAK-981 (SUMOylation inhibitor), MLN-4924 (NEDDylation inhibitor), and Bortezomib (Proteasome inhibitor), chemically synthesized compounds. TRBP2-immunodetection profile is lost (white arrows) only in the dividing cells undergoing mitosis, in contrast to the interphase cells. LX-2 cells treated with 1 µM DMSO were used as control. Green color: TRBP2; Red color: α-Tubulin; Blue color: Nucleus (DAPI). Scale bars: 10 µm. Inserts indicate aberrant mitoses being derived from each inhibitor’s pathogenic actions.

To unveil the molecular mechanism that orchestrates the loss of TRBP2 expression during mitosis, we reasoned to chemically target, essential for cell viability, post-translational modification (PTM) processes, with SUMOylation, NEDDylation, and Ubiquitination being characteristic examples. Hence, the three different inhibitors TAK-981, MLN-4924, and Bortezomib, which can specifically target the SUMOylation-, NEDDylation-, and Proteasome-dependent [and, thus, the Ubiquitin–Proteasome System (UPS)] activities, respectively, were herein used. LX-2 hepatic cells were exposed to each one of these inhibitors (1 µM) at a time for 24 h and, subsequently, processed for immunofluorescence assays. LX-2 cells being treated with DMSO (1 µM, 24 h) only were used as a suitable control. Importantly, none of the three inhibitors proved able to cause any alteration in the TRBP2-immunodetection profile during cell division, compared to control settings, as the interphase-specific TRBP2 nuclear-compartmentalization signal is missing from the LX-2 dividing cells undergoing mitosis, independently of the presence or absence of each respective inhibitor (TAK-981, MLN-4924, and Bortezomib) herein examined (Figure 10).

Similarly, PACT distribution and expression were shown to remain unaffected in LX-2 cells being exposed to TAK-981, MLN-4924, or Bortezomib inhibitors, respectively, since PACT protein exhibited a pattern of cytoplasmic compartmentalization during interphase and a dispersed, in both the cytoplasm and nucleus, pattern at the mitosis stage, regardless of the presence or absence of each inhibitor (Supplementary Figure S3). Of note, the nuclear detection of ATF4 transcription factor exclusively in the inhibitor-treated LX-2 cells (Supplementary Figure S4) indicated the activation of the ATF4-signaling branch, which belongs to the endoplasmic reticulum (ER)-stress network, in response to TAK-981, MLN-4924, or Bortezomib inhibitor administration, thereby confirming inhibitors’ efficacies to induce LX-2 hepatic-cell responses and pathologies. Furthermore, the impact of each inhibitor on cell proliferation was examined by measuring the percentage of mitotic-cell number in the presence or absence of the inhibitor (TAK-981, MLN-4924, or Bortezomib). Interestingly, targeted inhibition of SUMOylation-, NEDDylation-, or Proteasome-dependent processes seemed to alter proliferation rates, with mitotic-cell percentage changing from ~11.6% (control: absence of inhibitor) to ~14.7% (TAK-981), ~7.3% (MLN-4924), and ~9.9% (Bortezomib) (Supplementary Figure S5), further corroborating each inhibitor’s proficiency to trigger cell-division disturbances in LX-2 hepatic-cell environments.

Taken together, we herein reveal that SUMOylation-, NEDDylation- and Proteasome-dependent mechanisms are not essential or play redundant/dispensable roles in the mitosis-specific loss of TRBP2 immunodetection and expression profiles. However, their inhibition is presented with significant perturbations in mitotic cell rate, thus indicating the critical contribution of SUMOylation, NEDDylation, or Ubiquitination (UPS) pathways to hepatic cell cycle regulation in a TRBP2-independent manner.

### 3.11. ERK-, JNK-, p38 MAPK-, and AKT-Signaling Activities Are Not Required for TRBP2 Downregulation During Mitosis in LX-2 Dividing Cells

Given that protein phosphorylation is considered a fundamental and widespread type of PTM in mammalian cells, we next aimed at chemically inhibiting critical kinase family members, with ERKs, JNKs, p38 MAPK, and AKTs being representative examples. Therefore, to investigate if TRBP2 could be subjected to protein phosphorylation during interphase and/or mitosis, MEK1/2 (they signal upstream of their ERK1/2 bona fide substrates), JNK1/2/3, p38 MAPK, and AKT1/2/3 serine/threonine protein kinases were chemically targeted and functionally inhibited by exposure of LX-2 (hepatic) cells to the respective protein kinase inhibitors U0126 (100 µM; MEK1/2), SP600125 (50 µM; JNK1/2/3), SB203580 (100 µM; p38 MAPK), and MK-2206 (25 µM; AKT1/2/3), all being used at clinically relevant doses (for 24 h) able to cause notable pathologies (e.g., apoptotic features) in LX-2-treated cells [Supplementary Figure S6 (inserts) and Material intended for publication]. LX-2 hepatic cells, having been exposed to the appropriate DMSO concentrations for 24 h, served as controls.

**Figure 11:**
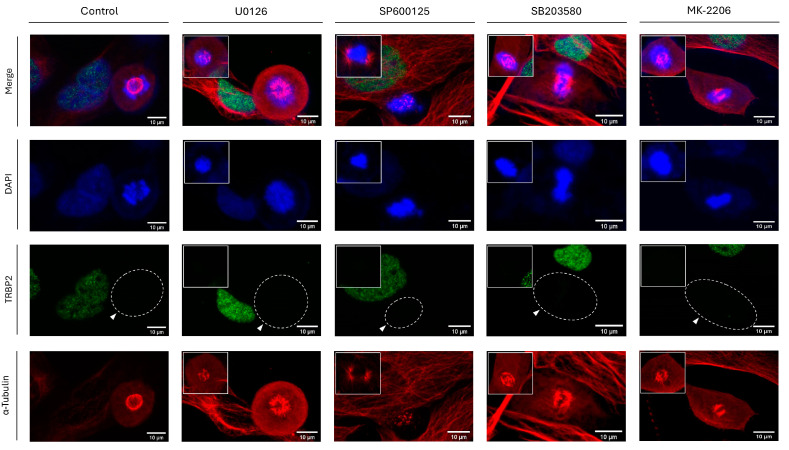
Chemical inhibition of major signaling pathways cannot rescue the TRBP2 protein-specific loss during mitosis in the LX-2 dividing cell. Immunofluorescence images of LX-2 cells, exploring for TRBP2 immunostaining patterns in interphase and mitotic cells, 24 h after the administration of 100 µM U0126 [MEK1/2 (ERK1/2) inhibitor], 50 µM SP600125 (JNK1/2/3 inhibitor), 100 µM SB203580 (p38 MAPK inhibitor) and 25 µM MK-2206 (AKT1/2/3 inhibitor). DMSO was used as a control condition. TRBP2 protein immunodetection pattern is missing (white arrows) only in the dividing cells undergoing mitosis, in contrast to the interphase cells. Green color: TRBP2; Red color: α-Tubulin; Blue color: Nucleus (DAPI). Scale bars: 10 µm. Inserts denote aberrant mitosis incidents, indicating the pathogenic efficacy of each chemical inhibitor.

Similar to SUMOylation, NEDDylation, and Proteasome activity inhibition (Figure 10), administration of U0126, SP600125, SB203580, or MK-2206 inhibitors in LX-2 dividing cells proved unable to change the immunostaining-mediated TRBP2-expression patterns, which were characterized by TRBP2 compartmentalization in the nucleus at the interphase stage and by TRBP2-detection loss during mitosis, either in the presence or in the absence of each inhibitor (U0126, SP600125, SB203580, and MK-2206) herein used (Figure 11). Likewise, no inhibitor-induced alteration could be recognized for PACT compartmentalization since the protein was detected mainly in the cytoplasm at the interphase, while it was dispersed all over each (LX-2) cell at mitosis, regardless of the respective inhibitor’s actions (Supplementary Figure S6). Altogether, it seems that none of the MEK-ERK-, JNK-, p38 MAPK-, and AKT-signaling axes can essentially control TRBP2 downregulation (loss) during mitosis, although a signaling-redundancy process among them cannot be excluded.

### 3.12. Microtubule-Network Disruption Cannot Affect TRBP2-Expression Profile During Mitosis in LX-2 Hepatic Cells

**Figure 12:**
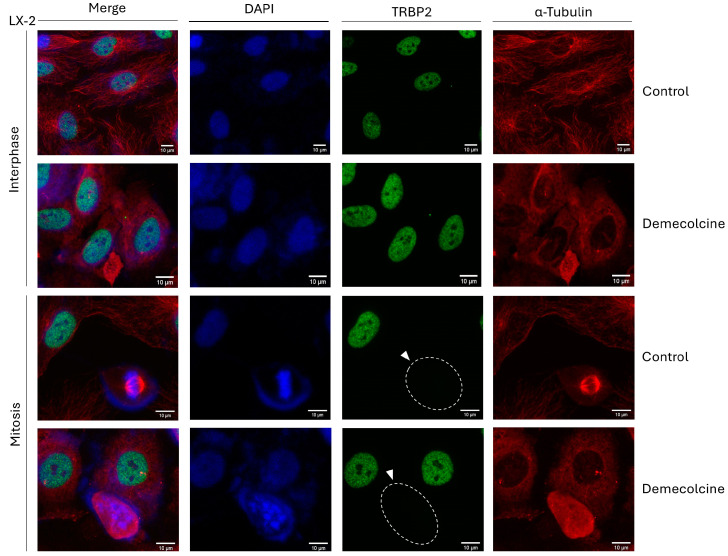
Microtubule-network disruption cannot restore the mitosis-specific lack of TRBP2-immunodetection pattern in LX-2 hepatic cells. Immunofluorescence images of LX-2 cells, investigating for TRBP2 expression, after 6 h treatment with the microtubule-polymerization inhibitor Demecolcine (0.4 µg/µL), in interphase (**upper panels**) and mitotic (**lower panels**) cells. TRBP2 protein is missing from mitotic cells (white arrows), both in treated (Demecolcine) and control (DMSO) conditions, in contrast to interphase cells. Green color: TRBP2; Red color: α-Tubulin; Blue color: Nucleus (DAPI). Scale bars: 10 µm.

Since the loss of TRBP2 protein is observed exclusively at the mitosis stage of cell cycle, which is typified by a dramatic re-organization of the microtubule-based cytoskeleton, to timely assemble the mitotic spindle, we, next, reasoned to chemically disrupt the microtubule-network integrity, seeking for alterations in TRBP2′s immunodetection profiles, during cell division. Thereby, LX-2 dividing cells were exposed to Demecolcine (0.4 µg/µL), a specific inhibitor of microtubule polymerization, for 6 h, and cells were, subsequently, processed for immunofluorescence-facilitated imaging of TRBP2-protein expression and distribution. Interestingly, despite the Demecolcine-induced disruption of microtubule-dependent cytoskeleton (Figure 12 and Supplementary Figure S7), TRBP2 could retain its interphase-specific nuclear compartmentalization, which was completely lost at the mitosis stage, in the presence of Demecolcine, exhibiting remarkably similar phenotypes to the control (DMSO only) settings (Figure 12). Of note, Demecolcine did not seem to have an effect on PACT’s protein expression and distribution, either at the interphase (cytoplasmic pattern) or at the mitosis (dispersed pattern) stage of the LX-2 dividing cells (Supplementary Figure S7). Taken together, it is, herein, revealed that TRBP2 protein does not depend on microtubule-cytoskeleton integrity and mitotic-spindle formation, for its mitosis-specific loss during (LX-2) hepatic-cell division, proliferation, and growth.

### 3.13. Mapping of the Cell Compartment-Specific TRBP2 Molecular Interactome in Human-Disease Settings

**Figure 13:**
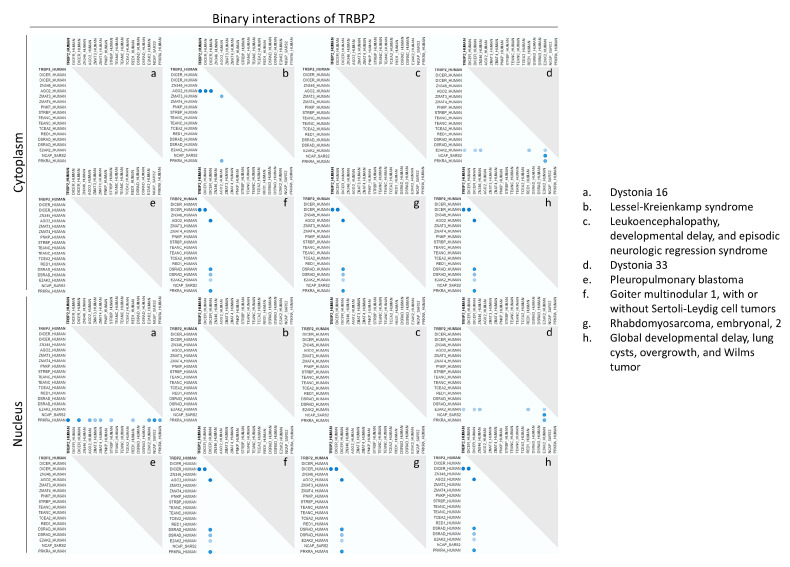
Cell compartment-specific TRBP2 interactome in human diseases. Dot plots graphically displaying major binary interactions of TRBP2 protein in different sub-cellular compartments, and especially cytoplasm (**upper panels**) and nucleus (**lower panels**), in diverse human diseases, including cancer (e.g., Wilms tumor) (**a**–**h**).

In an effort to mechanistically comprehend TRBP2′s role in cell division control, we in silico analyzed the binary interactions of TRBP2 protein in different sub-cellular compartments and diverse human pathologies. Disease-specific patterns of TRBP2 interactions could be herein recognized, with DICER being unveiled as the major TRBP2 partner (Figure 13). Remarkably, different sub-cellular compartments were presented to accommodate distinct TRBP2 interactomes, as was clearly demonstrated by the identification of TRBP2-AGO2 interaction in the cytoplasm but not in the nucleus of Lessel–Kreienkamp (syndrome) cells. Reversely, a TRBP2-PRKRA/PACT interaction could be detected in the nucleus, but not in the cytoplasm, of Dystonia 16 (disorder) cells (Figure 13). Regarding PACT (PRKRA), a disease-dependent interaction between TRBP2 and DICER proteins was in silico described both in the cytoplasm and the nucleus, while PACT was presented with significantly enriched nuclear and cytoplasmic interactome profiles, as compared to the TRBP2 respective ones, in Dystonia 16 cells, likely rendering PACT a druggable target for disease’s therapy (Supplementary Figure S8). In accordance, a P222L mutation in the exon 7 of the *PRKRA* gene has been previously associated with a young-onset dystonia-parkinsonism disorder [61]. Furthermore, a PACT molecular interactome was detected in the cytoplasm, but not in the nucleus, of Leukoencephalopathy (disease) cells (Supplementary Figure S8), with the assembly of the PACT-PKR complex justifying the capacity of PACT to activate PKR protein kinase, as previously reported, specifically in the cytoplasm, during cell division. Importantly, Dystonia 16 cells were characterized by the presence of PACT and by the simultaneous absence of TRBP2 binary interactions in the cytoplasm, while Lessel–Kreienkamp cells were typified by similar respective patterns in the nucleus, strongly suggesting their (TRBP2 versus PACT) opposite functionalities in cell-cycle regulation mechanisms. Of note, the PACT interactome was shown to contain a larger number of components than the TRBP2 one in the Dystonia 16 cell nucleus, with the pathogenic cytoplasm lacking TRBP2 binary interactions, although being simultaneously enhanced with PACT respective ones.

Altogether, we strongly support the operation of a TRBP2/PACT-dependent dynamic interactome that can adjust its constituents in a cell compartment- and pathology-specific fashion to critically control cell proliferation and growth in human health and disease.

## 4. Discussion

TRBP2 protein, beyond its bona fide role in the RNAi-machinery control, has proved to be essentially involved in distinct, RNAi-independent molecular processes, with its seminal function being typified by the inhibition of PKR (interferon-inducible double-stranded RNA-activated protein kinase) substrate-phosphorylation activity. PKR is a ubiquitously expressed Serine/Threonine Kinase (STK) activated by dsRNAs [62,63,64], which can induce PKR to undergo dimerization and auto-phosphorylation, resulting in translational inhibition via phosphorylation of eIF-2A (α subunit of eukaryotic Initiation Factor 2), a major regulator of the translation apparatus [27,62]. Most importantly, TRBP2 can form a heterodimer with PKR in an RNA-independent manner, preventing its auto-phosphorylation and activation and promoting cell survival [26,27,49,65,66].

Furthermore, several studies indicate that TRBP2 plays an important role in diverse biological processes, including organism development, normal cell growth, and tissue pathology, such as cancer. During development, TRBP2 critically contributes to spermatogenesis and growth control, as TRBP2 loss causes morphological abnormalities, such as reduced body size and oligospermia in mice [21,37,67], while it also has an impact on neural stem-cell characteristics during brain development via activating the Notch signaling pathway in a DICER- and RNA-independent manner [68]. Notably, it has been demonstrated that TRBP2 can regulate angiogenesis, hypoxia-stress response, and chemoresistance in cancer [69,70,71]. Interestingly, novel, non-canonical, and direct roles for TRBP2 in gene expression regulation have emerged as well. TRBP2 can act as a *trans* factor that binds dsRNAs directly via specific structural *cis*-regulatory RNA elements, termed TRBP-binding structural elements (TBSE), in the 3΄-UTR of transcripts and destabilizes them [70,72,73]. Moreover, TRBP2 binding to pre-mRNAs into the nucleus leads to increased intron retention and degradation of these aberrant transcripts by the nuclear exosome [74].

Our results further extend these studies, given that the tight regulation of TRBP2-protein compartmentalization and expression seems to act as a major determinant of normal cell proliferation and growth. Apparently, TRBP2 levels are crucial for cell-cycle control, as has been shown herein by flow cytometry analysis and by the protein’s immunophenotypic loss or modification during mitosis that has proved mechanistically indispensable and cancer-type-wise universal. Importantly, *TARBP2* (coding gene for TRBP2) overexpression increased the LX-2 proliferation rate, thus dictating its cell-growth-promoting activity and subsequent presumed oncogenicity, with TRBP2 elimination at mitosis being absolutely required for the correct implementation of cell division, regardless of the initial protein levels during interphase. Taken together, TRBP2, due to its major contribution to cell division, must be subjected to strong regulatory mechanisms that can operate regardless of tissue, malignancy grade, metastatic, and molecular signature (mutational) profiles of tumor cells.

Due to the multi-functionality of the TRBP2 protein, its aberrant expression can lead to cell cycle dysfunction and, consequently, human pathology [19]. Hence, tight regulation of TRBP2 protein levels is required for normal cell division, proliferation, and growth. A common mechanism of TRBP2 regulation acts via proteasomal degradation that is being facilitated by Merlin. Merlin protein localizes at the membrane cytoskeleton during interphase and interacts with several proteins, inhibiting critical growth-signal pathways [42,75,76]. Among others, TRBP2 has been found as a novel protein directly interacting with Merlin through the 237–366 and 288–595 amino acid (aa) domains, located at their COOH-terminal ends, respectively [42,77]. Merlin binds to TRBP2 and promotes its Ubiquitination, thereby targeting TRBP2 to the proteasome for proteolytic degradation and elimination [37,42,69,77].

Another mechanism controlling TRBP2 protein levels is SUMOylation, with SUMO (Small Ubiquitin-related Modifier) representing a reversible protein modifier, which can be conjugated with several target substrates [78,79,80,81]. It has been previously reported that TRBP2 is SUMOylated at Lysine 52 (K52), increasing the gene-silencing efficiency of miRNAs via recruiting AGO2 but not influencing their biogenesis [78,79]. Most importantly, TRBP2 SUMOylation enhances protein’s stability, as it can reduce TRBP2 poly-Ubiquitination and prevent its degradation [78].

The Ubc9 protein is an essential component of SUMOylation since it not only provides activated SUMO but is also involved in the selection of many SUMO targets [80]. This specific selection is usually based on the recognition by Ubc9 of a consensus motif (“ΨKXE”; Ψ: a bulky aliphatic residue, typically L/I/V) in the target protein [82,83]. Surprisingly, by examining the TRBP2 protein sequence, the “ΨKXE” motif (“LKAE”) was indeed identified at the 51–54 aa position (https://www.ensembl.org/Homo_sapiens (accessed on 7 July 2024)), which prompted us to, next, proceed to constrained docking (e.g., molecularly modeled Protein–Protein Interaction (PPI) of high statistical confidence) in between Ubc9 and a “LKAE” motif-containing area of the TRBP2 protein. In addition, we carried out docking tests in between Ubc9 and each different domain of TRBP2 (one potential interaction at a time). Remarkably, using this motif area, the two proteins proved to be in silico interact with each other via several residues “bonding,” while it seems that there are also alternative binding sites in all three TRBP2 domains (DRBM1-DRBM3) (Supplementary Figures S9 and S10), which could potentially function regulatory.

Given the involvement of SUMOylation and Ubiquitin–Proteasome (UP) sub-routines in TRBP2 regulation, we considered the possibility that TRBP2 immunofluorescence profile loss during mitosis might implicate these two mechanisms. Furthermore, we also examined the importance of one more regulatory process, called NEDDylation, whose functional correlation with TRBP2 remains still elusive. The NEDDylation pathway uses the Ubiquitin-like protein NEDD8, which is essential for the enzymatic activity of a subclass of Ubiquitin E3 Ligases (mediate for the movement of Ubiquitin from its carrier to the protein substrate) with NEDDylation enabling Ubiquitination and degradation rates of proteins, several of which have been found to control either normal cellular function or cancer cell development [84,85].

However, chemical inhibition of the three pathways does not seem to have an impact on TRBP2 immunophenotype, with mitotic cells lacking TRBP2 immunodetection either in the presence or in the absence of each inhibitor. Furthermore, measurements of mitotic-cell numbers indicate that the three pathways (SUMOylation, Ubiquitination, and NEDDylation) can affect cell division in a TRBP2-independent manner since their ability to alter dynamics of mitosis when they are inhibited cannot be coupled with the presumed re-appearance of TRBP2 immunofluorescence profile at the mitotic stage of the cell cycle. If we presume that SUMOylation inhibition can cause reduced TRBP2 protein levels, whereas Ubiquitination-Proteasome System (UPS) or NEDDylation inhibition may result in opposite effects (e.g., TRBP2 stabilization and upregulation), then (based on our *TARBP2*-overexpression data), cell-proliferation rates should decrease (in the SUMOylation-inhibition state) and increase (in the UPS- or NEDDylation-inhibition states), respectively. However, *TARBP2* (coding for TRBP2) overexpression leads to increased proliferation, which is in sharp contrast with the cellular responses of increased mitotic cell number upon SUMOylation inhibition-induced (presumable) reduction of the TRBP2 protein levels, thereby mechanistically uncoupling SUMOylation from TRBP2 expression loss during mitosis. Likewise, the expected elevation of TRBP2 contents in UPS- or NEDDylation-inhibited settings should enhance proliferation rates (according to our *TARBP2*-overexpression data), which, however, contradicts the reduced mitotic cell numbers observed (in the Bortezomib- or MLN-4924-driven responses). Altogether, we can conclude that TRBP2 protein is not subjected to any degradation process during cell division, prominently distinguishing two pathways for TRBP2 control: a mitotic-dependent (herein unveiled) and a mitotic-independent (described above) that may be functionally integrated in the regulation and dynamics of TRBP2 stability during cancer-cell life.

Phosphorylation on critical aa residues could effectively regulate TRBP2 functions as well. Recent reports have shown that TRBP2 is phosphorylated by two MAP kinases (MAPKs), the ERK(1/2) and JNK(1/2/3) ones, in response to oxidative stress or/and during mitosis [26,48]. Specifically, ERK-mediated phosphorylation of TRBP2 protein enhances growth-promoting miRNA production by increasing RISC stability, as the phosphorylation leads to a stronger interaction between TRBP2 and DICER [27,86]. On the other hand, JNK-mediated phosphorylation of TRBP2 affects its interaction with PKR and, consequently, PKR activation [27,48,86]. Interestingly, phosphorylation of TRBP2 by ERKs seems to enhance the protein’s SUMOylation that causes repression of its Ubiquitination and ensuing TRBP2 stabilization, strongly upregulating RNAi-system’s efficiency [78]. However, neither ERKs and JNKs nor p38 MAPK and AKT(1/2/3) Serine/Threonine Kinases can decisively act on TRBP2 immunophenotype during cell division, and especially mitosis since their chemical inhibition proved unable to restore the mitosis-specific loss of TRBP2 immunodetection, herein described for the first time. It seems that mitosis-dependent (our study) and mitosis-independent (previous studies) signaling networks act differently on TRBP2 protein, thereby unveiling its multifaceted and versatile roles in tightly regulating cancer cell diverse functions during division, migration, metastasis, and chemoresistance.

The employment of an in silico approach seems to essentially contribute to a better understanding of the TRBP2 novel functionalities in cell cycle control. Docking tests of TRBP2 protein with major cell cycle regulators revealed significant interaction potentials with Cyclin-A1 and CDK2 proteins, as well as the Cyclin-E1 protein, while its (TRBP2) binding to the Ubc9 protein, albeit with comparatively lower “docking values” (e.g., quantification of dissociation constants that characterize intrinsic structural stabilities of protein-containing, bi-molecular complexes) (Supplementary Figure S11), strongly suggested a new role for the TRBP2-Ubc9 complex (besides TRBP2 SUMOylation) in mitosis dynamics. Since Cyclin-A1 acts as a key cell-cycle regulator, necessary for the entry into S and M phases, and can form a complex (among others) with CDK2 kinase, enhancing its interaction with critical substrates [87,88], the TRBP2 protein may serve as a novel, important target for phosphorylation by the activated CDK2 kinase, and this phosphorylation may function catalytically in cell cycle progression and cell division. Cyclin-E1 is also a fundamental cell cycle regulator, which is required for the entry into the S phase and can form a complex with CDK2 as well [89]. The increased levels of this complex at the G1/S transition phase are in line with the increased contents of TRBP2 protein at the same stage being observed by our flow cytometry analysis, strongly supporting their mechanistic correlation during the cell cycle.

In the same direction and searching for recorded interactors of TRBP2 and PACT proteins (Supplementary Tables S1 and S2), we distinguished KIF2A (Kinesin Family Member 2A), a plus end-directed microtubule-dependent motor with de-polymerization activity [90], and IQGAP2 (IQ Motif Containing GTPase Activating Protein 2), a Ras GTPase-activating protein [91], as unique TRBP2 interactors of presumably major importance (Supplementary Table S3). Docking tests unveiled the significant potential (low dissociation constant) for strong interactions between the components of both TRBP2-KIF2A and TRBP2-IQGAP2 complexes (Supplementary Figure S11). Given the pivotal role of KIF2A in mitotic-spindle formation and normal spindle dynamics during mitosis [90,92], together with the involvement of IQGAP2 in the regulation of cell morphology and motility and cancer development [91], the presumed interactions of TRBP2 with KIF2A and IQGAP2 proteins further support our current molecular model for the negative control of TRBP2 on KIF2A and IQGAP2 activities during cell division and specific stages of tumor initiation, progression, and metastasis. Altogether, it is the loss of TRBP2 protein that releases KIF2A and IQGAP2 functionalities during mitosis of dividing cells.

Regarding Protein–Protein Interactions (PPIs), they constitute a dynamic network that can change according to the phase of the cell cycle and the needs of the cell. Disruption in any PPI profile may result in pathological conditions and the development of human diseases. Indeed, by observing a wide range of human pathologies, we can detect critical changes in TRBP2 and PACT interactions, both among diseases and in between sub-cellular compartments (e.g., cytoplasm versus nucleus) (Figure 13 and Supplementary Figure S8). Remarkably, both TRBP2 and PACT proteins were, herein, in silico shown to interact with the DICER protein, regardless of the examined sub-cellular compartment (cytoplasm versus nucleus), in Goiter multinodular 1, Rhabdomyosarcoma, embryonal, 2 and Wilms tumor diseases being related to DICER1 syndrome [93,94,95,96], while they also interact with the AGO2 protein in Lessel–Kreienkamp syndrome (although in distinct sub-cellular compartments), a neuro-developmental disorder related to *AGO2* gene mutations [97]. Of note, TRBP2 and PACT can interact with AGO2 only in the cytoplasm and the nucleus, respectively, thus emphasizing the importance of each interaction in health and disease. This is also the case for Dystonia 16, a disorder caused by *PACT/PRKRA* gene mutations [98], with TRBP2 losing its ability to interact with PACT in the cytoplasm (retained in the nucleus) but with PACT gaining several interactors, including TRBP2, both in the cytoplasm and the nucleus as well.

Taken all together, TRBP2 is herein highlighted as an important regulator of the cell cycle, following an expression pattern similar to the ones of known cell-cycle regulators. Its interaction with such types of major regulatory proteins could be an issue of fundamental importance for normal cell division and growth. Notably, it seems that the roles of TRBP2 in a cell are not limited to the RNAi mechanism but are rather extended to diverse processes, and thus, its tight regulation is vital for cell function and fate. Given the number of mutations or aberrant expression patterns of TRBP2 that have been previously associated with human pathologies, such as cardiomyopathy, ADHD (Attention Deficit Hyperactivity Disorder), Alzheimer’s disease [99,100,101], and cancer [45,102,103,104,105,106], together with its (TRBP2) herein described, for the first time, new role(s) in mitosis of dividing cells, TRBP2 protein is likely rendered a promising prognostic, diagnostic, and therapeutic—druggable—biomarker for various human diseases, with the further investigation of its mitosis-controlling mechanism(s) most probably opening new therapeutic windows for diverse malignancies, either at the primary or at the metastatic stage(s).

## 5. Conclusions

Through the employment of advanced imaging, we herein demonstrate the nuclear compartmentalization of TRBP2 protein during interphase and its complete loss at the mitotic stage of dividing cells. Our findings dictate a negative role of TRBP2 in cell cycle control, thereby rendering its mitosis-specific restoration as an innovative, dynamic, and promising tool for genetic therapy of human cancer in the clinic. Nevertheless, more strenuous efforts must be made to productively overcome the existing difficulties in obtaining functional antibodies, working in both Western blotting and immunofluorescence technologies, and, also, being able to discriminate the two protein isoforms, thereby facilitating their subsequent quantitative assessment that is required for the molecular dissection of mitotic machinery, in the presence or absence of the TRBP2 regulator. Given the inherent complexity of cell division, the lack of TRBP2 immunodetection in mitotic cells is necessitated to be mechanistically illuminated as a new RNAi-independent sub-routine towards the identification of novel and druggable biomarkers for diverse malignancies.

## Data Availability

All data are available in the main text or Supplementary Materials.

## Supplementary Materials

The following supporting information can be downloaded at: https://www.mdpi.com/article/10.3390/cancers16213701/s1, Figure S1: In silico dissection of TRBP2, TRBP1, and PACT molecular structures: from gene to protein; Figure S2: T24-specific gating course for cell cycle phase partitioning; Figure S3: PACT-specific immunophenotypic profiles in hepatic cells undergoing SUMOylation, NEDDylation, or Proteasome inhibition-induced stress; Figure S4: Chemical inhibition of SUMOylation, NEDDylation, or UPS sub-routines induces ATF4-dependent activation of endoplasmic reticulum (ER) stress in hepatic cells; Figure S5: Targeted inhibition of SUMOylation, NEDDylation, or Proteasomal-degradation activities causes irregularities in mitotic rates of hepatic cells; Figure S6: Functional disintegration of major signaling pathways proves unable to alter PACT-specific immunophenotypes in hepatic cells; Figure S7: Chemical poisoning of microtubule cytoskeleton dynamics cannot affect PACT expression and compartmentalization patterns in hepatic cells; Figure S8: In silico mapping of PACT-specific interactomes in cell compartments and pathology-dependent manners; Figure S9: Molecular modeling of TRBP2 and Ubc9 interaction; Figure S10: In silico identification of the critical amino acid (aa) residues that mediate the TRBP2 and Ubc9 bi-molecular interaction; Figure S11: Bioinformatic quantification of molecular complex stabilities via measurements of dissociation constants between TRBP2 and major components of the cell cycle machinery; Table S1: TRBP2 interactors; Table S2: PACT interactors; Table S3: TRBP2-specific and unique interactors.
