# Supplementary material for: TRBP2, a Major Component of the RNAi Machinery, Is Subjected to Cell Cycle-Dependent Regulation in Human Cancer Cells of Diverse Tissue Origin
Source: Cancers (Basel). 2024 Nov 1;16(21):3701. doi: 10.3390/cancers16213701 (PMC11545598; doi:10.3390/cancers16213701)
Supplement: Supplementary file 1 [file cancers-16-03701-s001.zip › Theotoki_et al_October_Cancers_Supplementary_Materials_2024.pdf]

cancers

MDPI

Article - Supplementary Materials

TRBP2, a Major Component of the RNAi Machinery, Is Subjected to Cell Cycle-Dependent Regulation in Human Cancer Cells of Diverse Tissue Origin

Eleni I. Theotoki [1,2], Panos Kakoulidis [2,3], Athanassios D. Velentzas [1], Konstantinos-Stylianos Nikolakopoulos [1], Nikolaos V. Angelis [4], Ourania E. Tsitsilonis [4], Ema Anastasiadou [2,5,*,†] and Dimitrios J. Stravopodis [1,*,†]

[1] Section of Cell Biology and Biophysics, Department of Biology, School of Science, National and Kapodistrian University of Athens (NKUA), 157 01 Athens, Greece; elthk@biol.uoa.gr (E.I.T.); tveletz@biol.uoa.gr (A.D.V.); ksnikolakop@biol.uoa.gr (K.-S.N.)
[2] Center of Basic Research, Biomedical Research Foundation of the Academy of Athens (BRFAA), 115 27 Athens, Greece; pkakoulidis@di.uoa.gr
[3] Department of Informatics and Telecommunications, School of Science, National and Kapodistrian University of Athens (NKUA), 157 01 Athens, Greece
[4] Section of Animal and Human Physiology, Department of Biology, School of Science, National and Kapodistrian University of Athens (NKUA), 157 01 Athens, Greece; nangelis@biol.uoa.gr (N.V.A.); rtsitsil@biol.uoa.gr (O.E.T.)
[5] Department of Health Science, Higher Colleges of Technology (HCT), Academic City Campus, Dubai 17155, United Arab Emirates
* Correspondence: anastasiadou@bioacademy.gr or eanastasiadou@hct.ac.ae (E.A.); dstravop@biol.uoa.gr (D.J.S.)
† These authors contributed equally to this work.

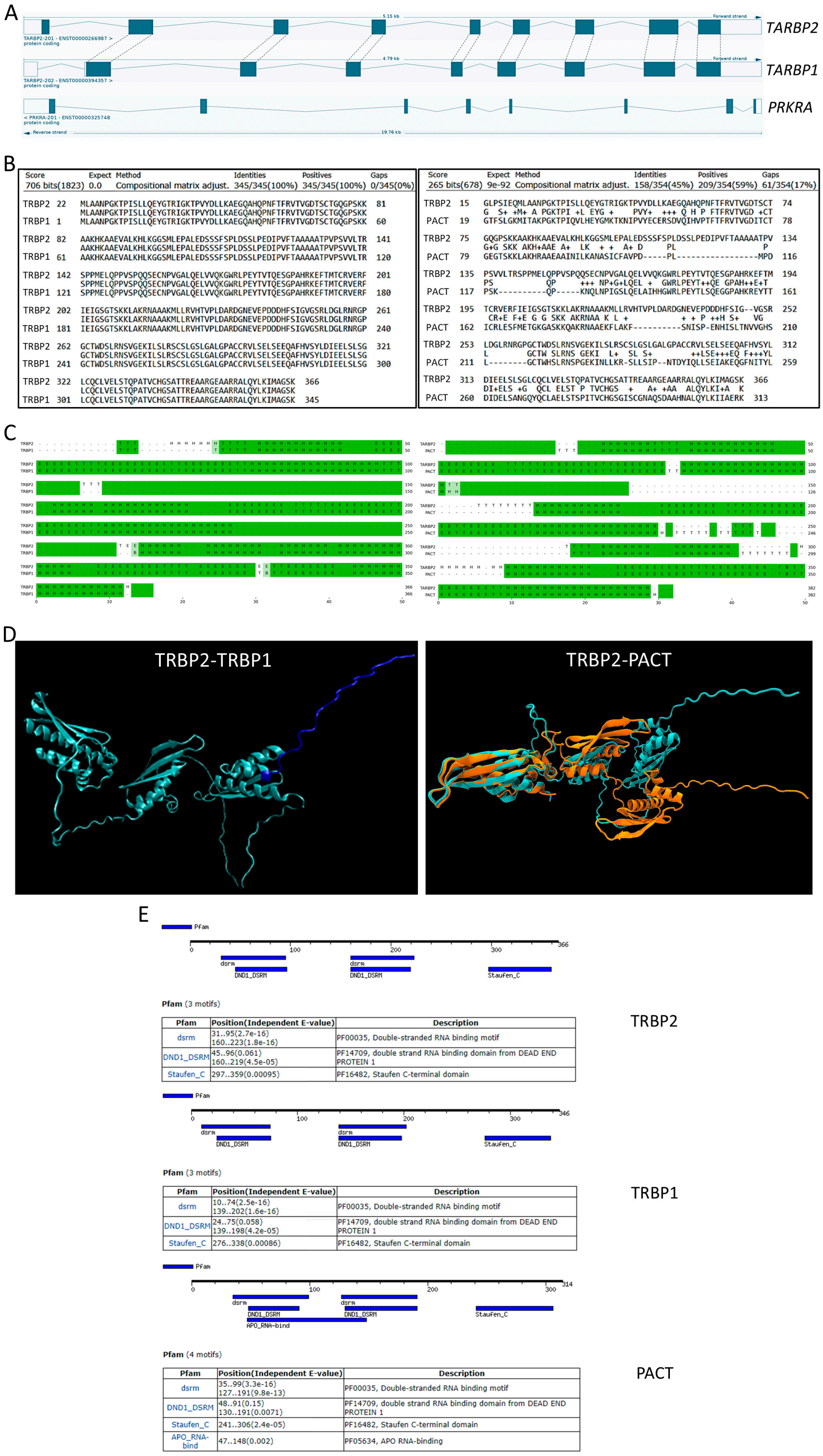

Supplementary Figure S1. ***In silico* dissection of TRBP2, TRBP1 and PACT molecular structures: from gene to protein.** (**A**) Graphic presentation of *TARBP2*, *TARBP1* and *PRKRA/PACT* transcripts, as they were obtained from the "Ensembl Genome Browser 113" (https://www.ensembl.org/Homo_sapiens/Info/Index), with exons and introns being clearly distinguished and aligned. Similar exons in between *TARBP2* and *TARBP1* transcript species are marked with black dashed lines. (**B**) Protein sequence "Blast-Alignment" in between TRBP2-TRBP1 (left panel) and TRBP2-PACT (right panel). (**C**) Secondary structure (2D) -in silico- comparison in between TRBP2-TRBP1 and TRBP2-PACT proteins. (**D**) Super-position of TRBP2-TRBP1 and TRBP2-PACT tertiary -*in silico*- structures (3D). In TRBP2, the protein segment missing from TRBP1 is marked (blue ribbon). (**E**) Motif map of the TRBP2 (top), TRBP1 (middle) and PACT (bottom) proteins, via employment of the "Motif Search" suite ("MOTIF: Searching Protein Sequence Motifs") (https://www.genome.jp/tools/motif/).

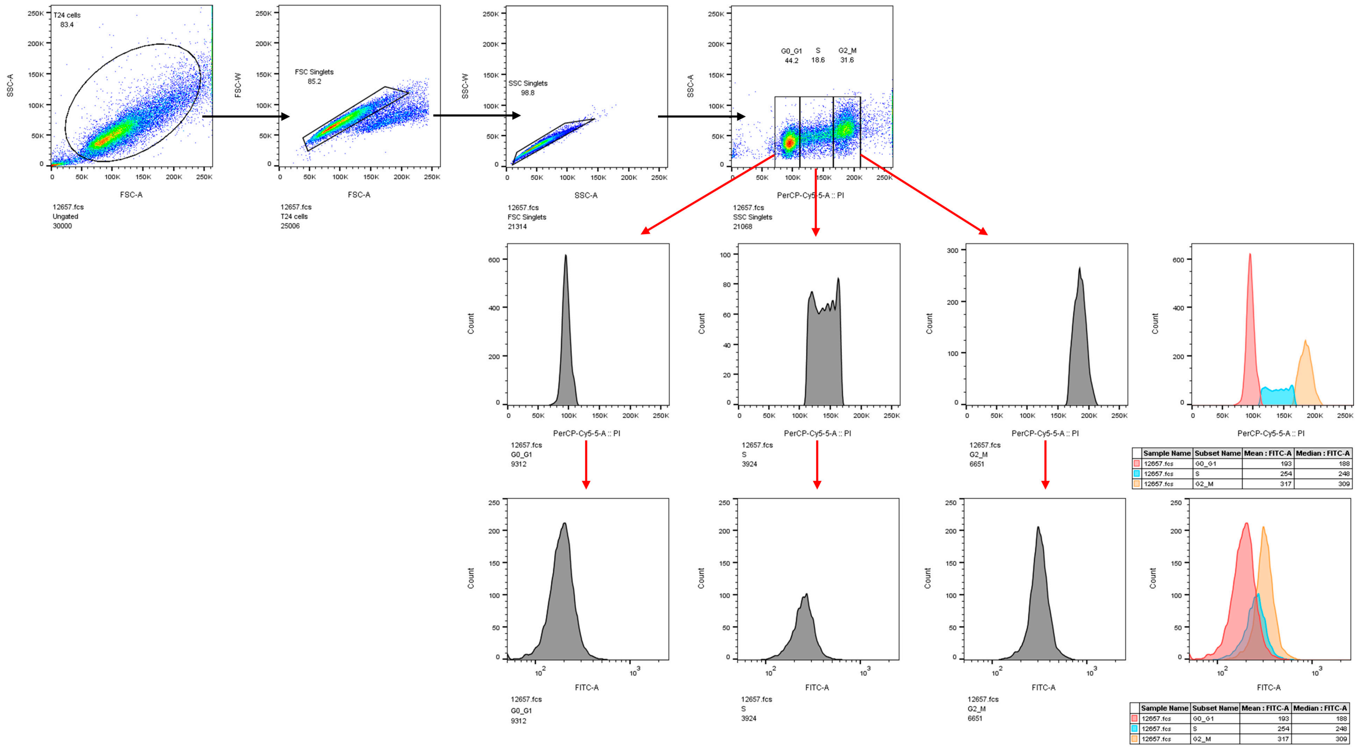

Supplementary Figure S2. T24-specific gating course for cell cycle-phase partitioning. Gating strategy for the detection of FITC-positive cells at the G0/G1, S and G2/M phases of the cell cycle, in T24 dividing cells.

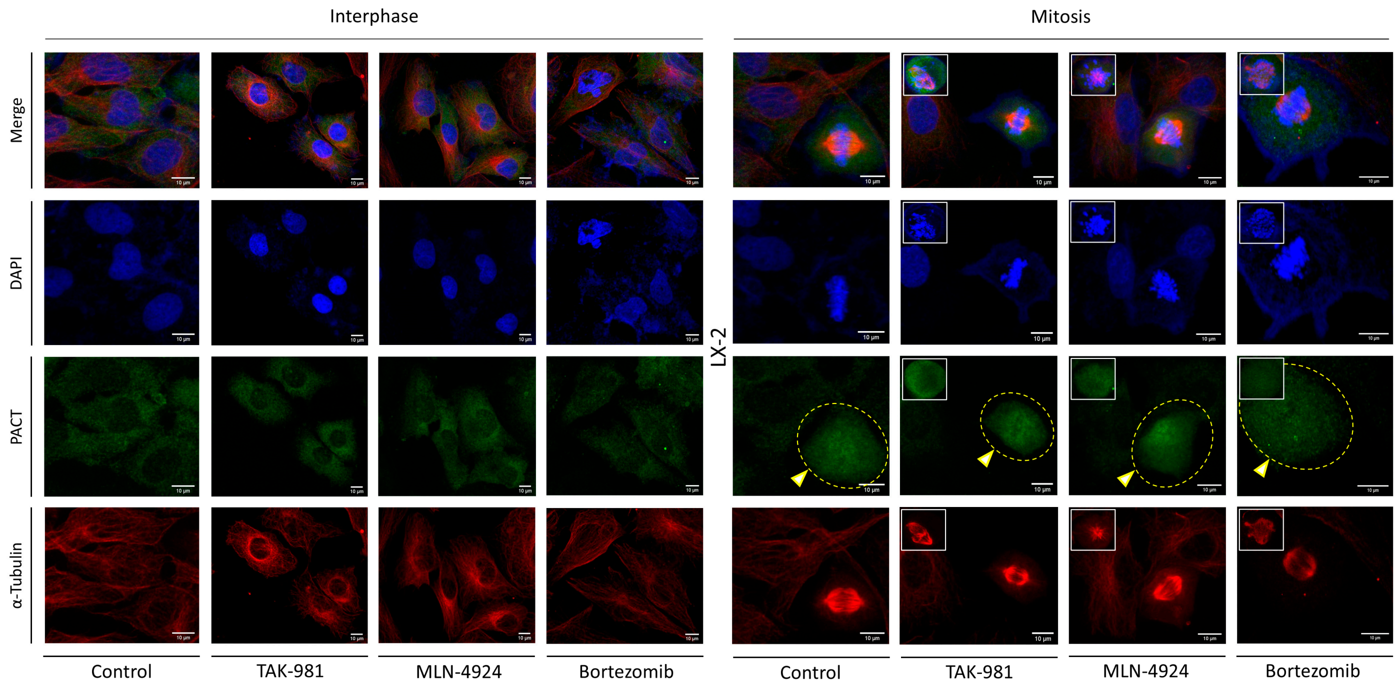

Supplementary Figure S3. PACT-specific immunophenotypic profiles in hepatic cells undergoing SUMOylation, NEDDylation, or Proteasome inhibition-induced stress. Immunofluorescence images of LX-2 cells, exhibiting PACT expression in interphase (left panels) and mitotic (right panels) cells, 24 h after administration of 1 μM TAK-981 (SUMOylation inhibitor), MLN-4924 (NEDDylation inhibitor) and Bortezomib (Proteasome inhibitor) synthetic compounds. Note that PACT protein is expressed in both interphase and dividing cells, undergoing mitosis (yellow arrows). LX-2 cells treated with 1 μM DMSO were used as control. Green color: PACT; Red color: α-Tubulin; Blue color: Nucleus (DAPI). Scale bars: 10 μm. Inserts illustrate aberrant-mitosis incidents, indicating the pathogenic activities of each, herein, administered inhibitor, in dividing LX-2 hepatic cells.

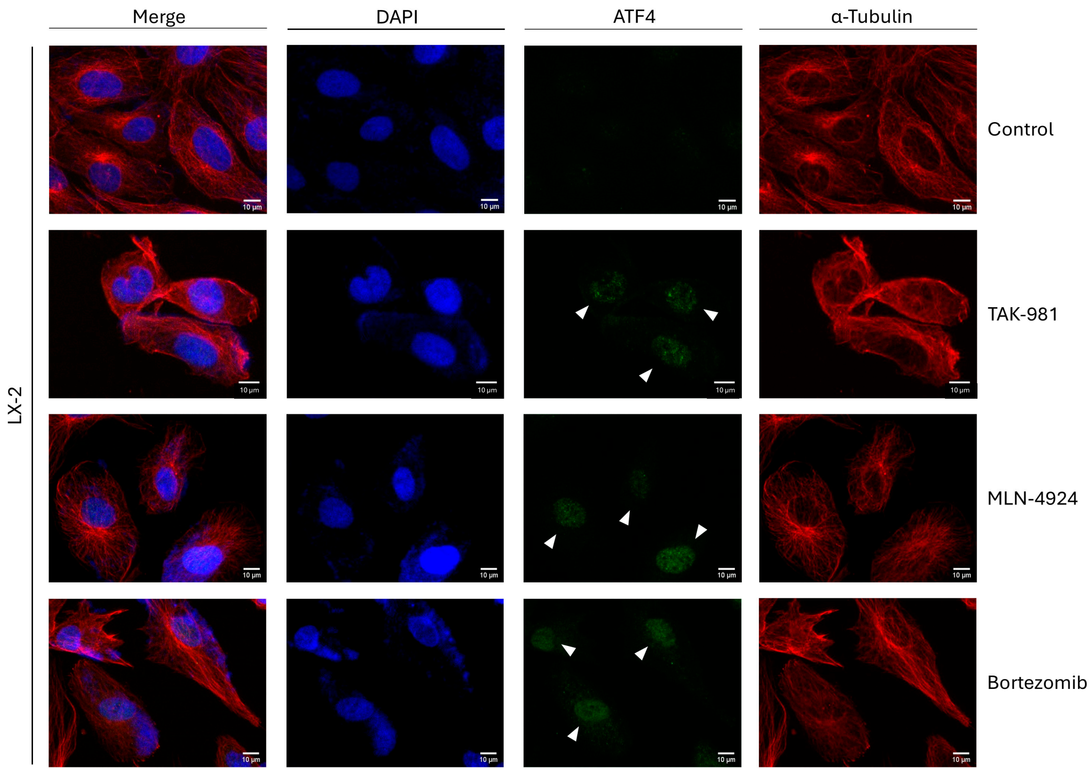

Supplementary Figure S4. Chemical inhibition of SUMOylation, NEDDylation, or UPS sub-routines induces ATF4-dependent activation of Endoplasmic-Reticulum (ER) stress in hepatic cells. Immunofluorescence images, illustrating the ATF4-protein immunodetection pattern in the nucleus of LX-2 cells (white arrows), 24 h after treatment with 1 μM TAK-981 (SUMOylation inhibitor), MLN-4924 (NEDDylation inhibitor), Bortezomib (Proteasome inhibitor) and DMSO (control). Green color: ATF4; Red color: α-Tubulin; Blue color: Nucleus (DAPI). Scale bars: 10 μm.

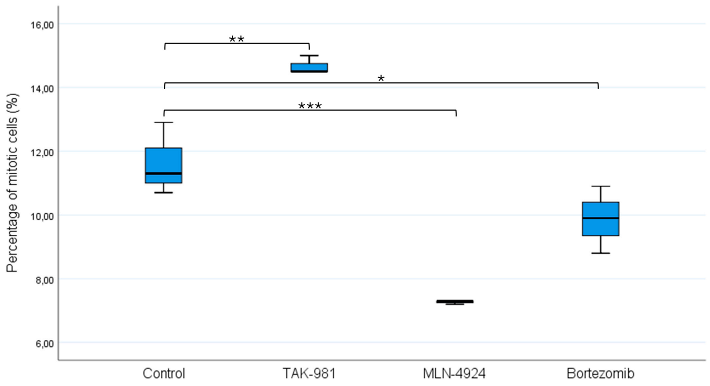

Supplementary Figure S5. Targeted inhibition of SUMOylation, NEDDylation, or Proteasomal-degradation activities causes irregularities in mitotic rates of hepatic cells. Box-plot graphically displaying the percentage of mitotic-cell number (%), 24 h after LX-2 cell exposure to 1 µM TAK-981 (SUMOylation inhibitor), MLN-4924 (NEDDylation inhibitor) and Bortezomib (Proteasome inhibitor) compounds, as compared to control cells (1 µM DMSO). * - ** - ***: p<0,05. Note that SUMOylation, NEDDylation and Proteasome inhibition can significantly affect proliferation rates of LX-2 hepatic cells.

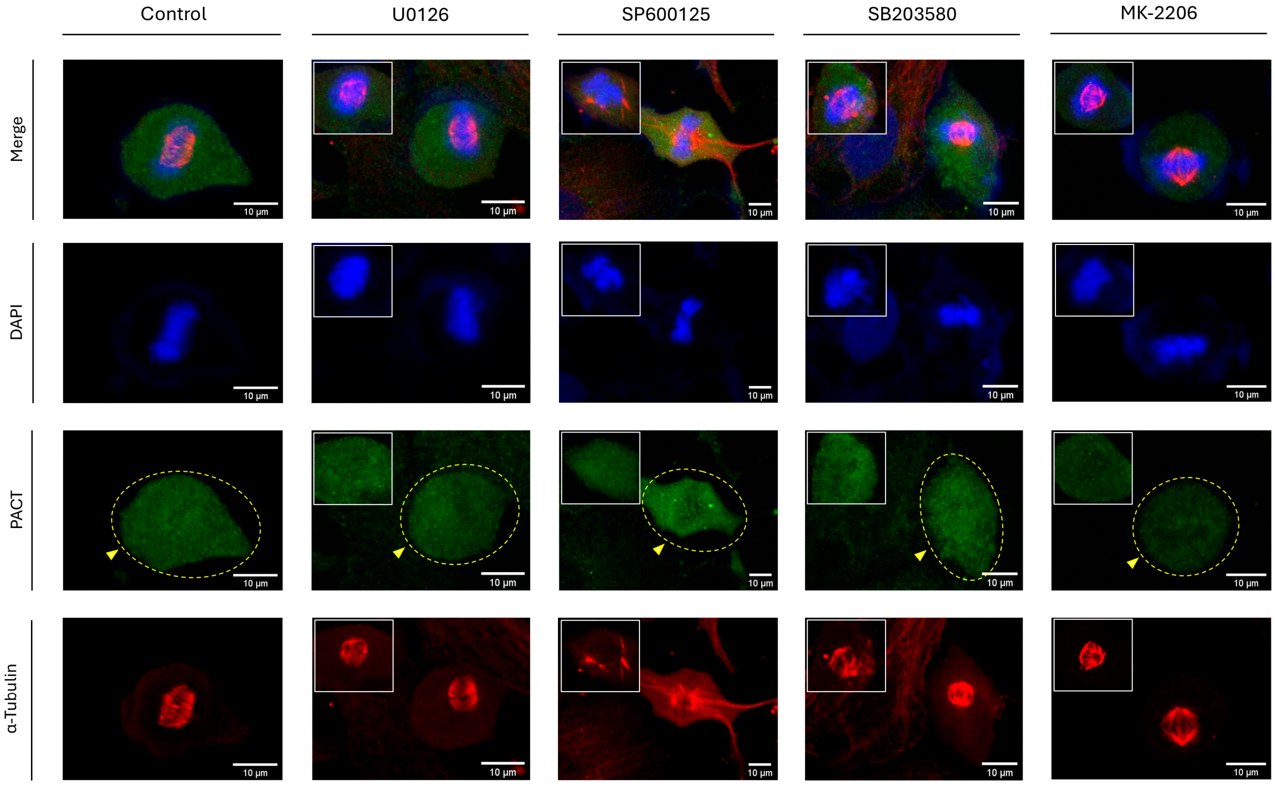

Supplementary Figure S6. Functional disintegration of major signaling pathways proves unable to alter PACT-specific immunophenotypes in hepatic cells. Immunofluorescence images of LX-2 cells, presenting PACT expression in mitotic cells, 24 h after treatment with 100 μM U0126 [MEKs (ERKs) inhibitor], 50 μM SP600125 (JNKs inhibitor), 100 μM SB203580 (p38 MAPK inhibitor) and 25 μM MK-2206 (AKTs inhibitor) chemical compounds. DMSO was used as control condition. Green color: PACT; Red color: α-Tubulin; Blue color: Nucleus (DAPI). Scale bars: 10 μm. Note that the chemical inhibition of critical signaling pathways cannot affect PACT protein-expression profile (yellow arrows), although aberrant mitoses are being formed in the presence of each inhibitor (inserts), strongly suggesting their (inhibitors') pathogenic actions in LX-2 dividing cells.

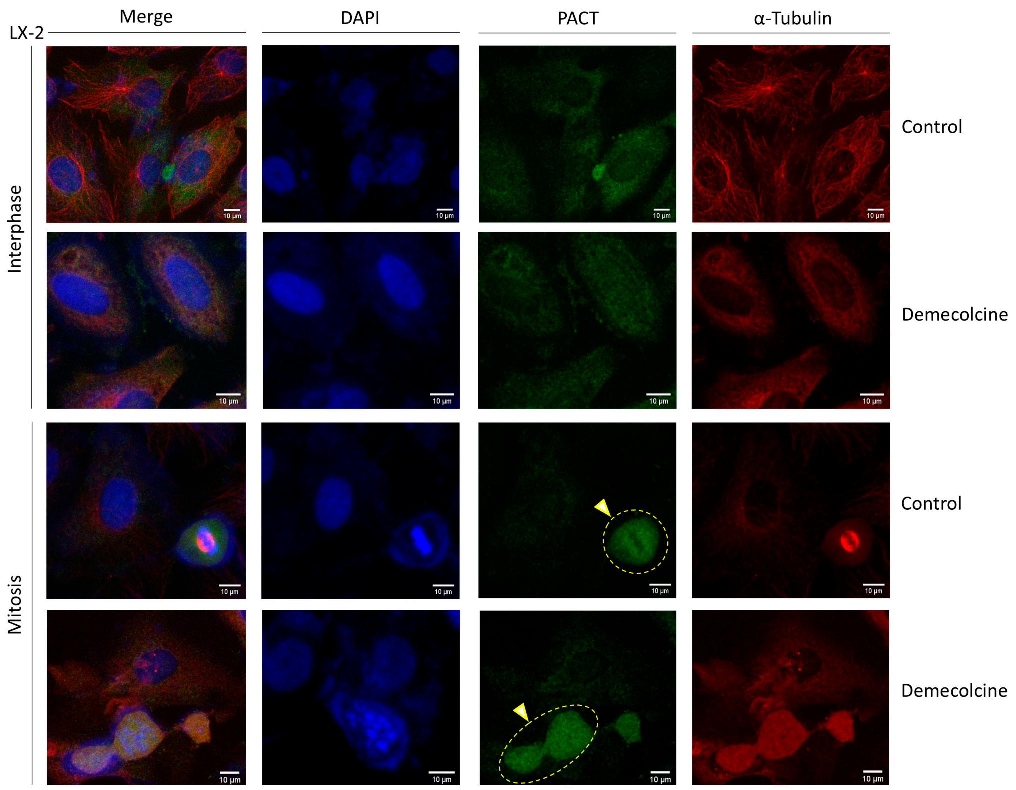

Supplementary Figure S7. Chemical poisoning of Microtubule-cytoskeleton dynamics cannot affect PACT-expression and -compartmentalization patterns in hepatic cells. Immunofluorescence images of LX-2 cells, depicting PACT expression and distribution, 6 h after administration of the Microtubule-polymerization inhibitor Demecolcine (0,4 μg/μL), in interphase (upper panels) and mitotic (lower panels) cells. PACT protein is expressed both in interphase and in dividing (mitotic) cells (yellow arrows), in inhibitor-treated and control (DMSO) cells. Green color: PACT; Red color: *α*-Tubulin; Blue color: Nucleus (DAPI). Scale bars: 10 μm. Note the Microtubule-network disruption in the Demecolcine-exposed LX-2 dividing (mitotic) cells.

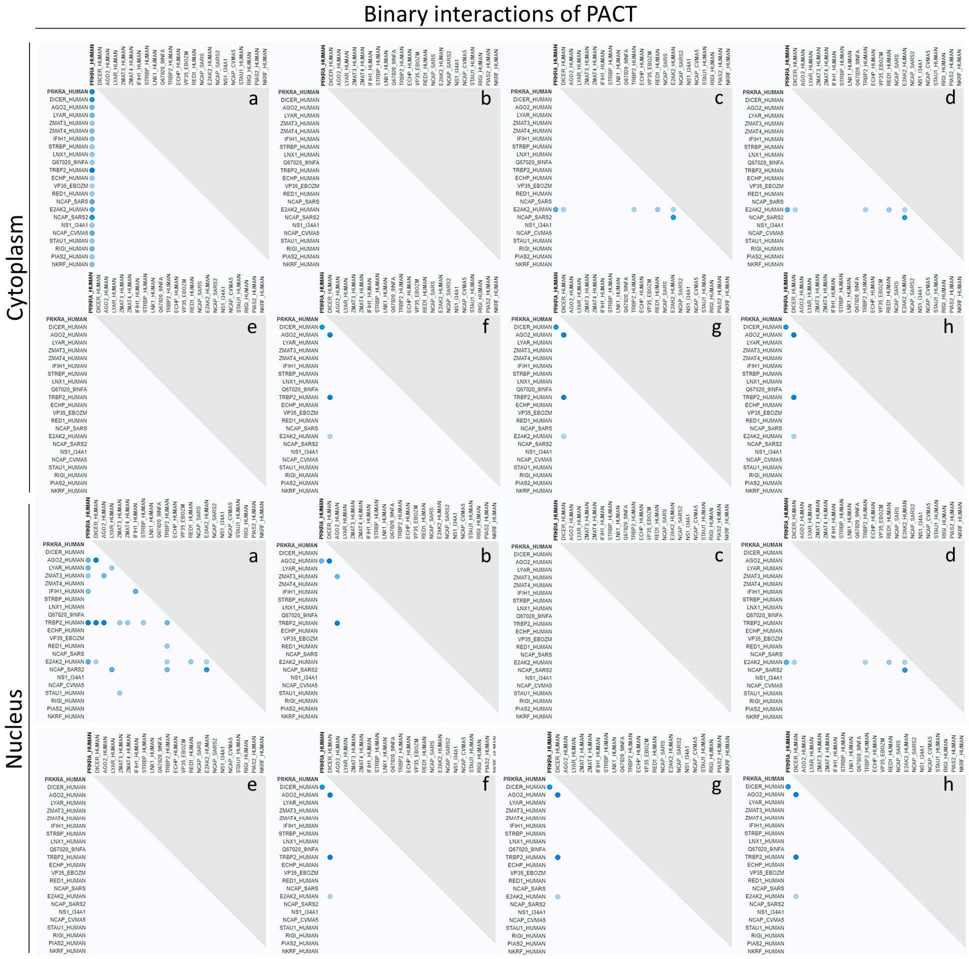

a. Dystonia 16
b. Lessel-Kreienkamp syndrome
c. Leukoencephalopathy, developmental delay, and episodic neurologic regression syndrome
d. Dystonia 33
e. Pleuropulmonary blastoma
f. Goiter multinodular 1, with or without Sertoli-Leydig cell tumors
g. Rhabdomyosarcoma, embryonal, 2
h. Global developmental delay, lung cysts, overgrowth, and Wilms tumor

Supplementary Figure S8. ***In silico*** **mapping of PACT-specific interactomes in cell compartment- and pathology-dependent manners.** Dot-blots graphically displaying binary interactions of PACT protein, in different sub-cellular compartments, and especially in the cytoplasm (upper panels) and the nucleus (lower panels), in diverse human diseases (a-h), including cancer (e.g., Wilms tumor). Note the distinct composition of components in between the TRBP2 (**Fig. 13**) and PACT (**Suppl. Fig. S8**) respective interactomes.

TRBP2-Ubc9 interaction

Chain	No. of interface residues	Interface area (Å^2)	No. of salt bridges	No. of disulphide bonds	No. of hydrogen bonds	No. of non-bonded contacts
A	9	498	1	-	7	71
B	9	503				

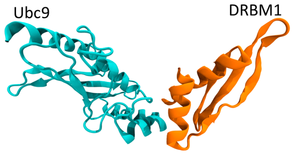

Chain	No. of interface residues	Interface area (Å^2)	No. of salt bridges	No. of disulphide bonds	No. of hydrogen bonds	No. of non-bonded contacts
A	12	681	-	-	12	97
B	12	648				

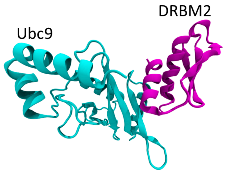

Chain	No. of interface residues	Interface area (Å^2)	No. of salt bridges	No. of disulphide bonds	No. of hydrogen bonds	No. of non-bonded contacts
A	11	546	3	-	4	62
B	12	539				

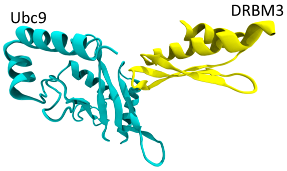

Chain	No. of interface residues	Interface area (Å^2)	No. of salt bridges	No. of disulphide bonds	No. of hydrogen bonds	No. of non-bonded contacts
A*	16	652	2	-	9	84
B*	12	684				

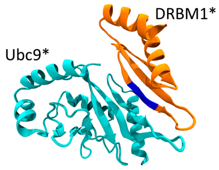

Supplementary Figure S9. Molecular modelling of TRBP2 and Ubc9 interaction. Docking tests in between each of the TRBP2, *in silico* predicted, main domains DRBM1, DRBM2 and DRBM3, with the Ubc9 protein. In the left-side panels, interface statistics of the interactions are indicated (chain A: Ubc9; chain B: TRBP2), while in the right-side panels, 3D structural models for the examined interactions are shown. Ubc9* - DRBM1*: Constrained docking with the [“LKAE”] (colored in blue) Ubc9-recognizing motif that is being localized at the 51-54 aa area of TRBP2 protein. Note the structural compatibility and complementarity in between the interacting components.

Interacting residues between TRBP2 and Ubc9 proteins		
**Protein**	**TRBP2 Domain**	**Amino acids**
TRBP2	DRBM1	33, 34, 37, 38, 41, 42, 95, 96, 97
	DRBM2	159, 162, 163, 166, 167, 169, 170, 222, 223, 224, 226, 227
	DRBM3	313, 314, 315, 316, 317, 318, 319, 320, 321, 342, 343, 344
	DRBM1*	47, 49, 50, 51, 52, 53, 54, 55, 57, 65, 67, 80
Ubc9	DRBM1	122, 125, 126, 134, 137, 138, 139, 141, 142
	DRBM2	13, 17, 20, 22, 23, 24, 25, 26, 27, 42, 57, 59
	DRBM3	13, 16, 17, 22, 24, 25, 27, 42, 57, 59, 157
	DRBM1*	13, 16, 17, 20, 21, 22, 23, 24, 25, 27, 28, 30, 34, 35, 36, 42

Supplementary Figure S10. *In silico* identification of the critical amino acid (aa) residues that mediate the TRBP2 and Ubc9 bi-molecular interaction. *In silico* protein-protein interactivity map, describing the potentially interacting aa residues in between the TRBP2 (DRBM1, DRBM2 and DRBM3) and Ubc9 proteins. DRBM1*: Constrained docking with the ["LKAE"] Ubc9-recognizing motif that resides at the 51-54 aa area of TRBP2 protein. Note the number of interacting aa residues involved.

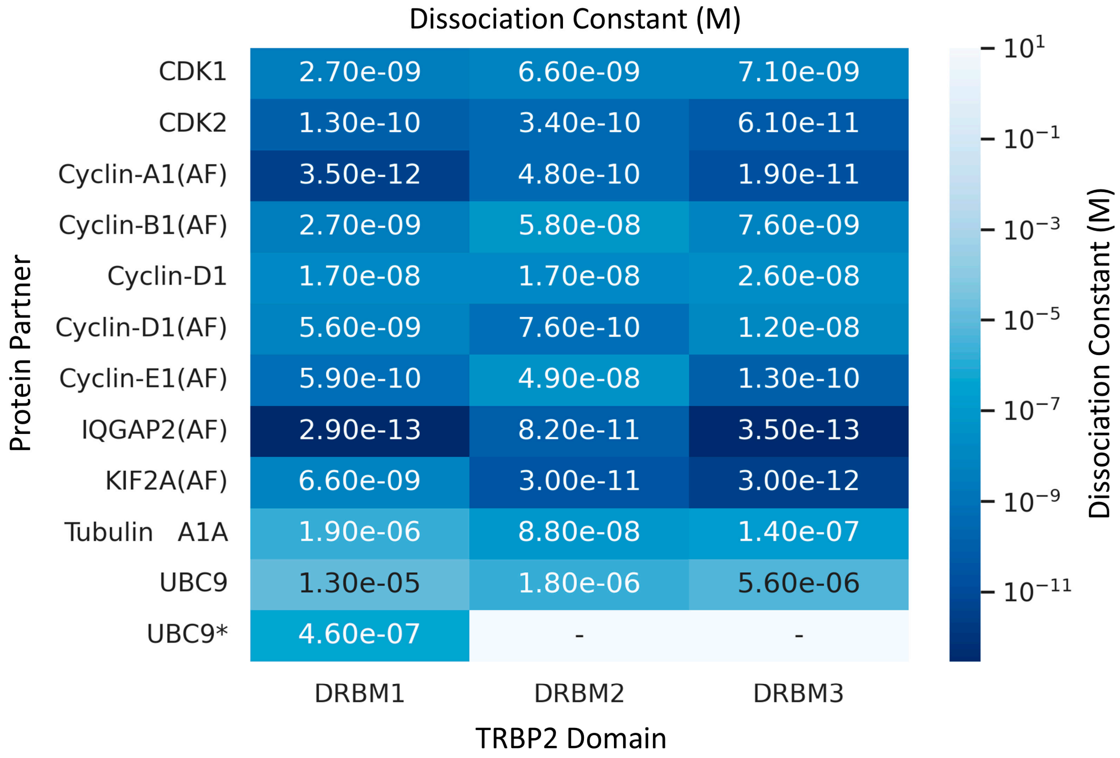

Supplementary Figure S11. Bioinformatic quantification of molecular-complex stabilities, via measurements of Dissociation Constants, in between TRBP2 and major components of the cell-cycle machinery. Docking tests in between each one of the three different TRBP2 domains (DRBM1, DRBM2 and DRBM3), with other proteins being critically implicated in cell-cycle control (AF: AlphaFold-mediated structural modelling), strongly supporting the validity of identified interactions. Ubc9*: Constrained docking with the [“LKAE”] Ubc9-recognizing motif that is being accommodated at the 51-54 aa area of TRBP2 protein. Note the remarkably low Dissociation-Constant (DC) values for Cyclins and CDKs, as putative TRBP2-protein partners and interactors.

Supplementary Table Captions (S1 – S3)

Supplementary Table S1. TRBP2-protein interactors, being derived from the BioGRID database

Supplementary Table S2. PACT-protein interactors, being derived from the BioGRID database

Supplementary Table S3. TRBP2-specific and unique interactors, as compared to the PACT protein-derived ones (Suppl. Tables S1 and S2 content subtraction)
