# Supplementary figures and images for: TRBP2, a Major Component of the RNAi Machinery, Is Subjected to Cell Cycle-Dependent Regulation in Human Cancer Cells of Diverse Tissue Origin
Source: Cancers (Basel). 2024 Nov 1;16(21):3701. doi: 10.3390/cancers16213701 (PMC11545598; doi:10.3390/cancers16213701)

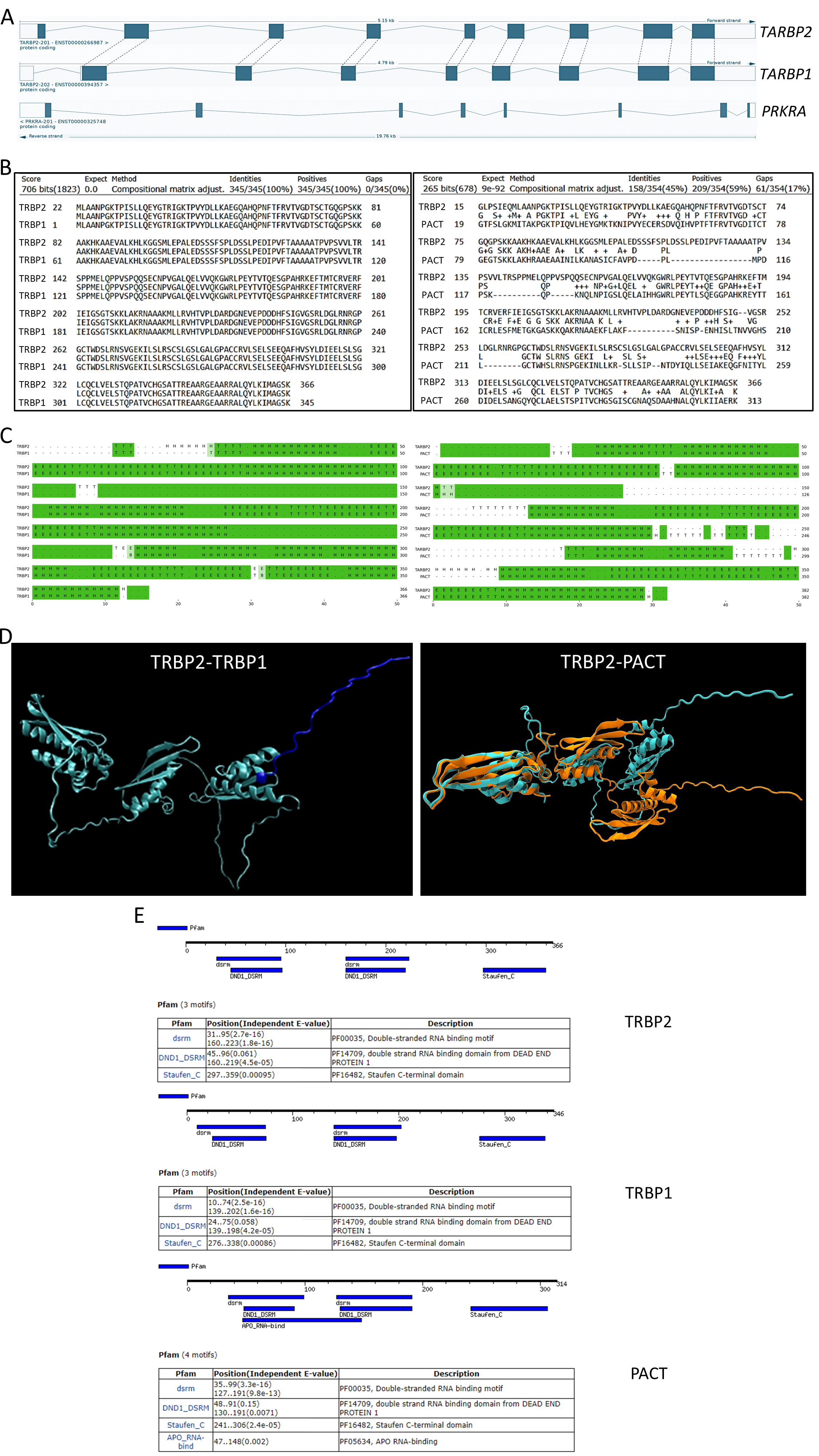

Supplement: Supplementary file 1 [file cancers-16-03701-s001.zip › S1.tif]

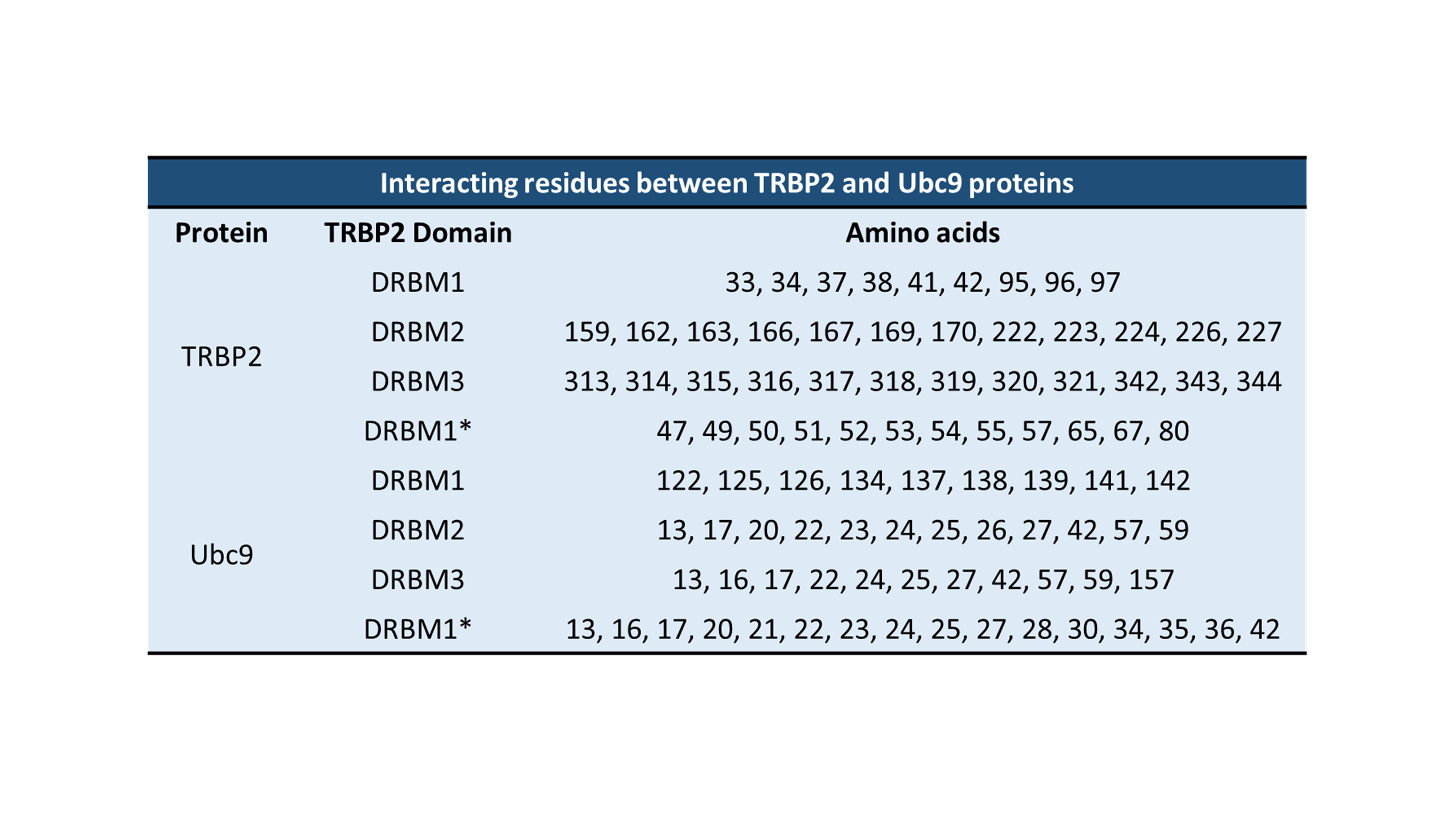

Supplement: Supplementary file 1 [file cancers-16-03701-s001.zip › S10.tif]

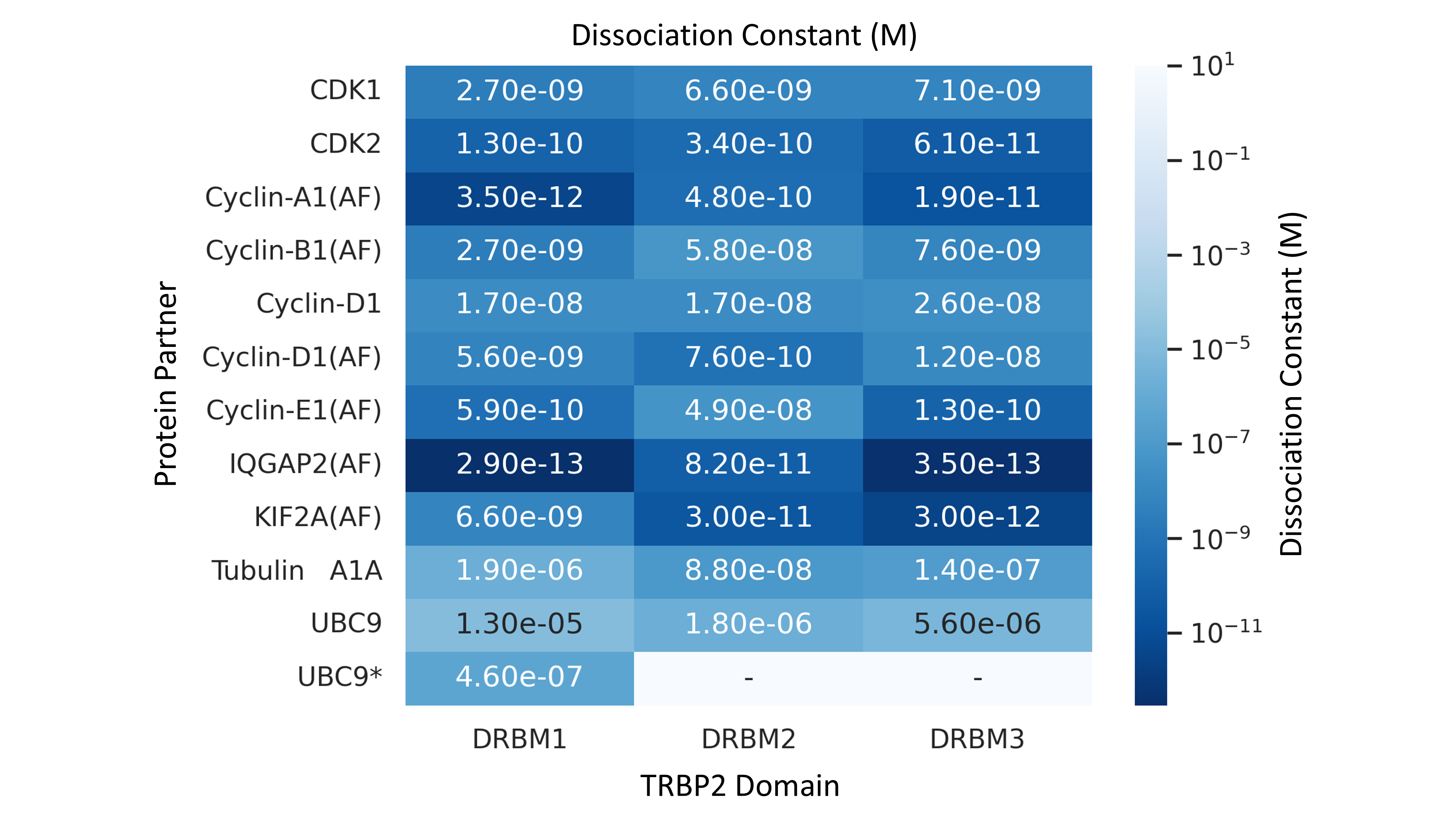

Supplement: Supplementary file 1 [file cancers-16-03701-s001.zip › S11.tif]

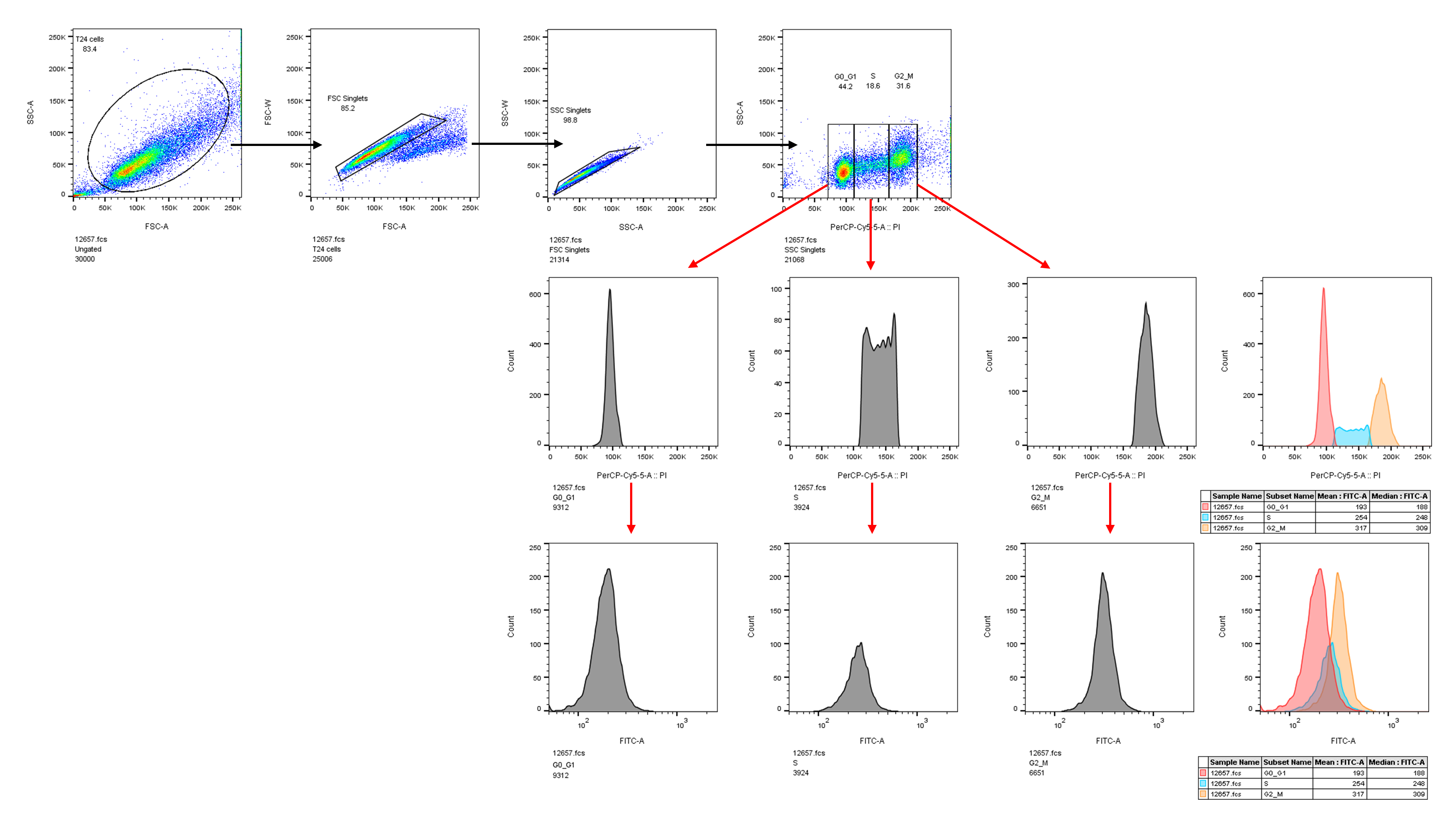

Supplement: Supplementary file 1 [file cancers-16-03701-s001.zip › S2.tif]

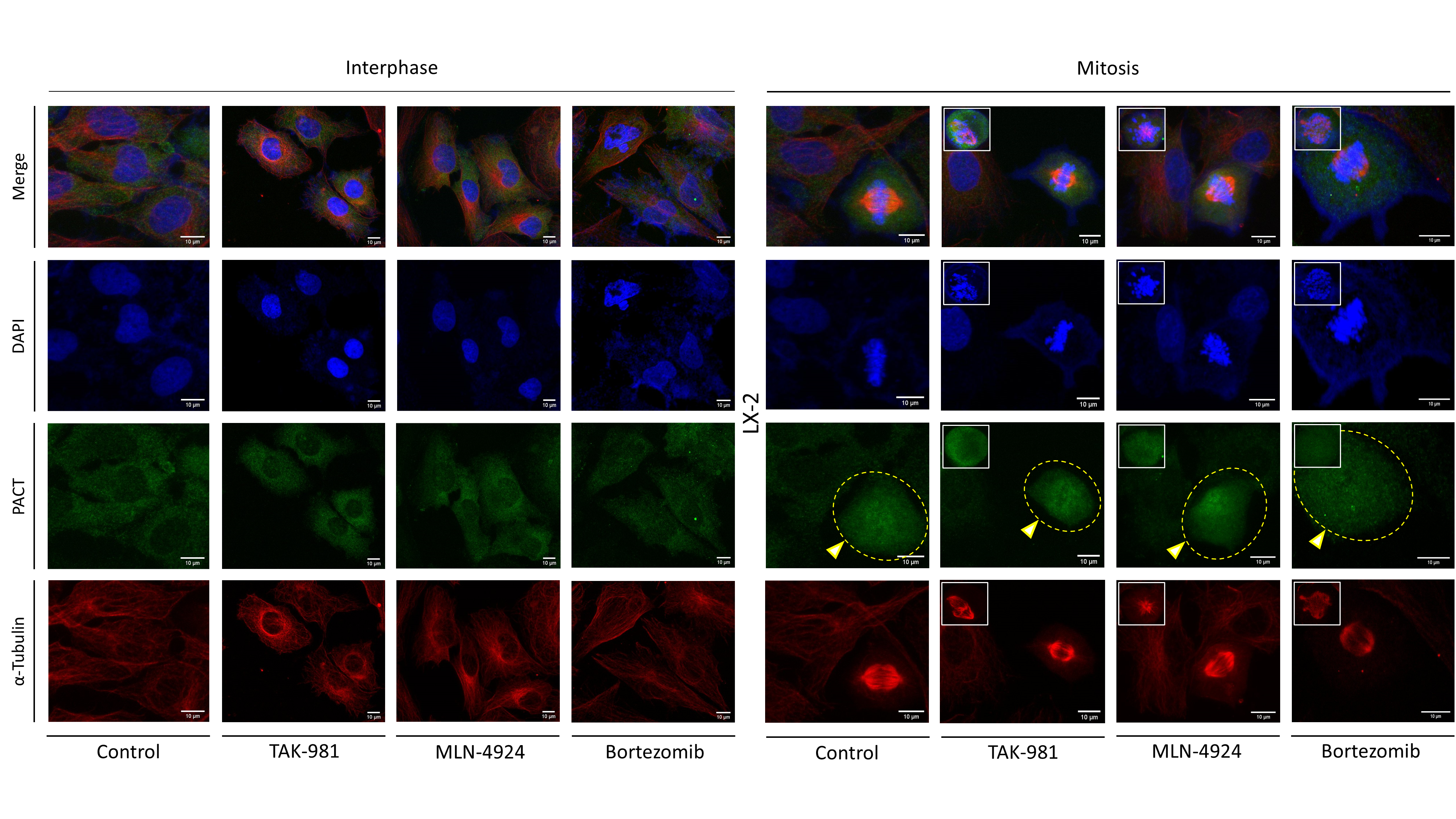

Supplement: Supplementary file 1 [file cancers-16-03701-s001.zip › S3.tif]

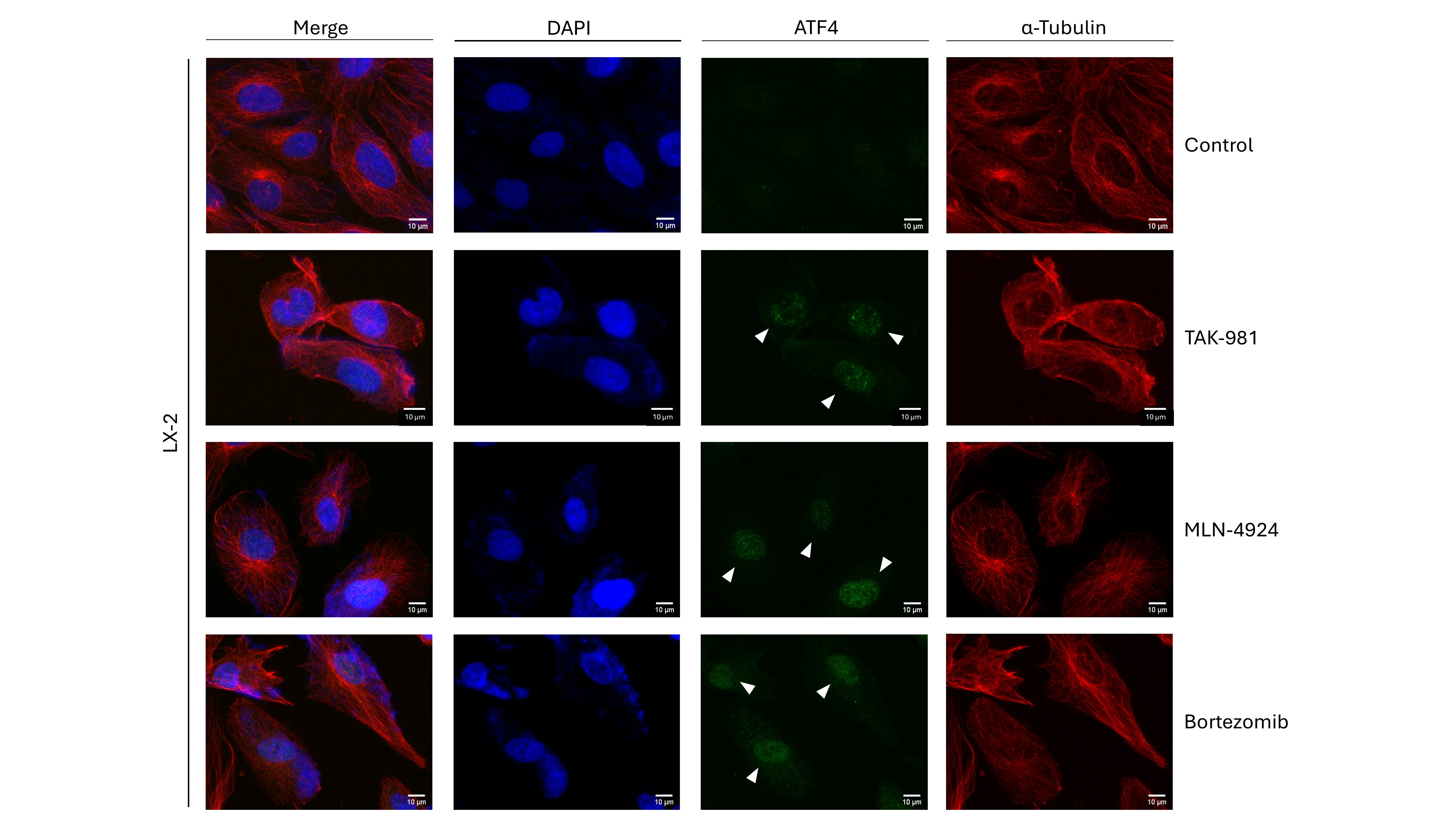

Supplement: Supplementary file 1 [file cancers-16-03701-s001.zip › S4.tif]

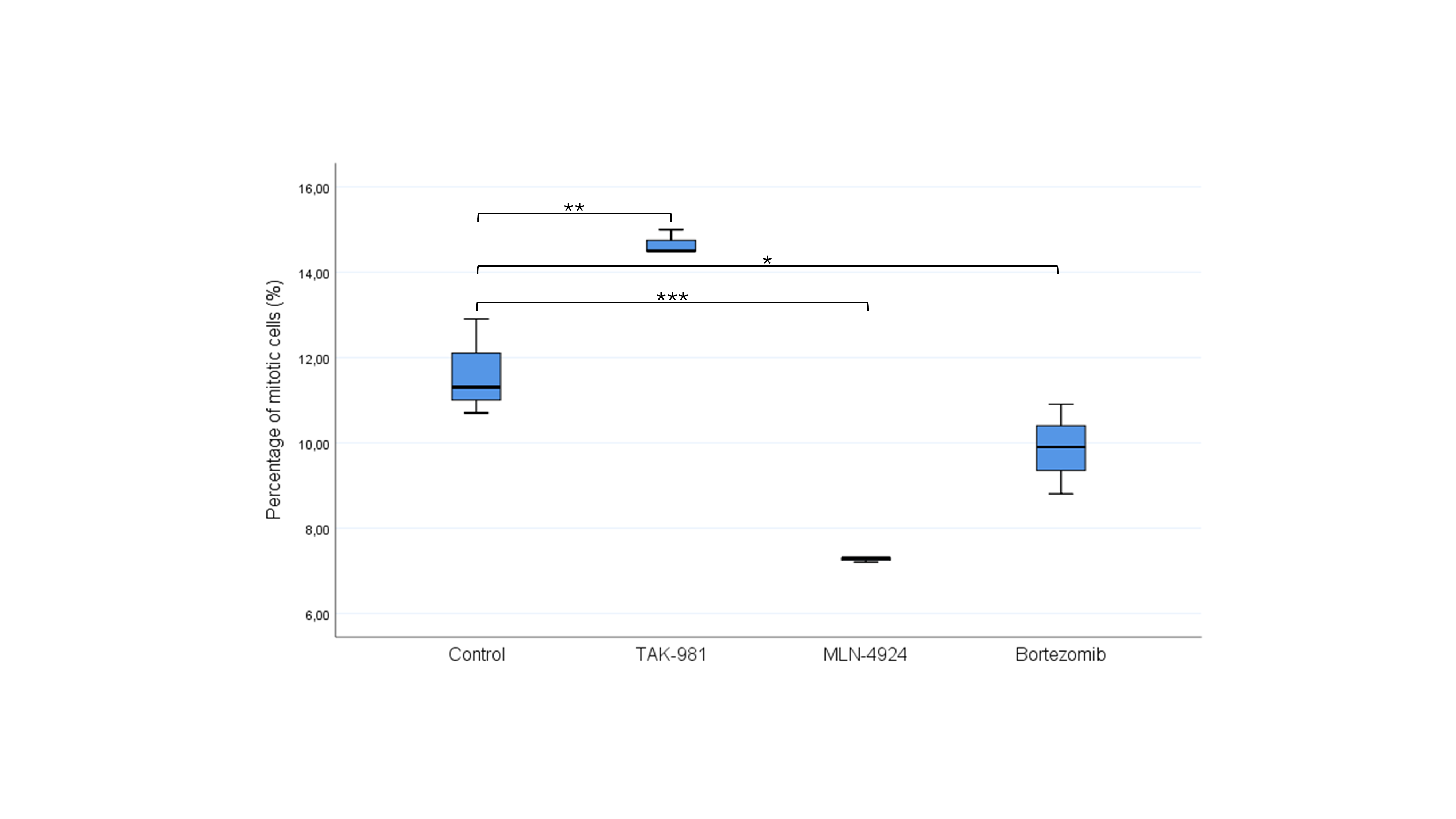

Supplement: Supplementary file 1 [file cancers-16-03701-s001.zip › S5.tif]

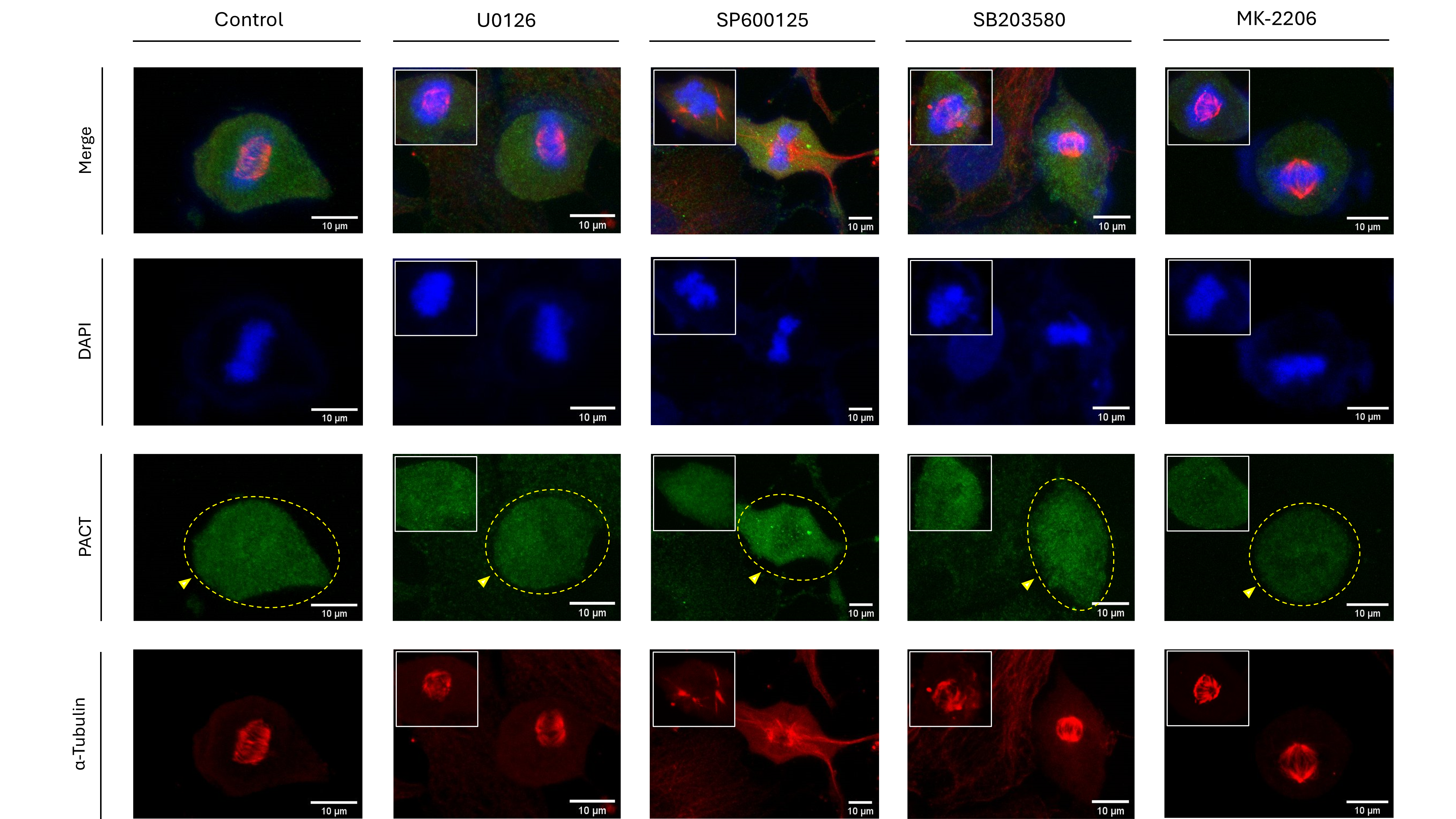

Supplement: Supplementary file 1 [file cancers-16-03701-s001.zip › S6.tif]

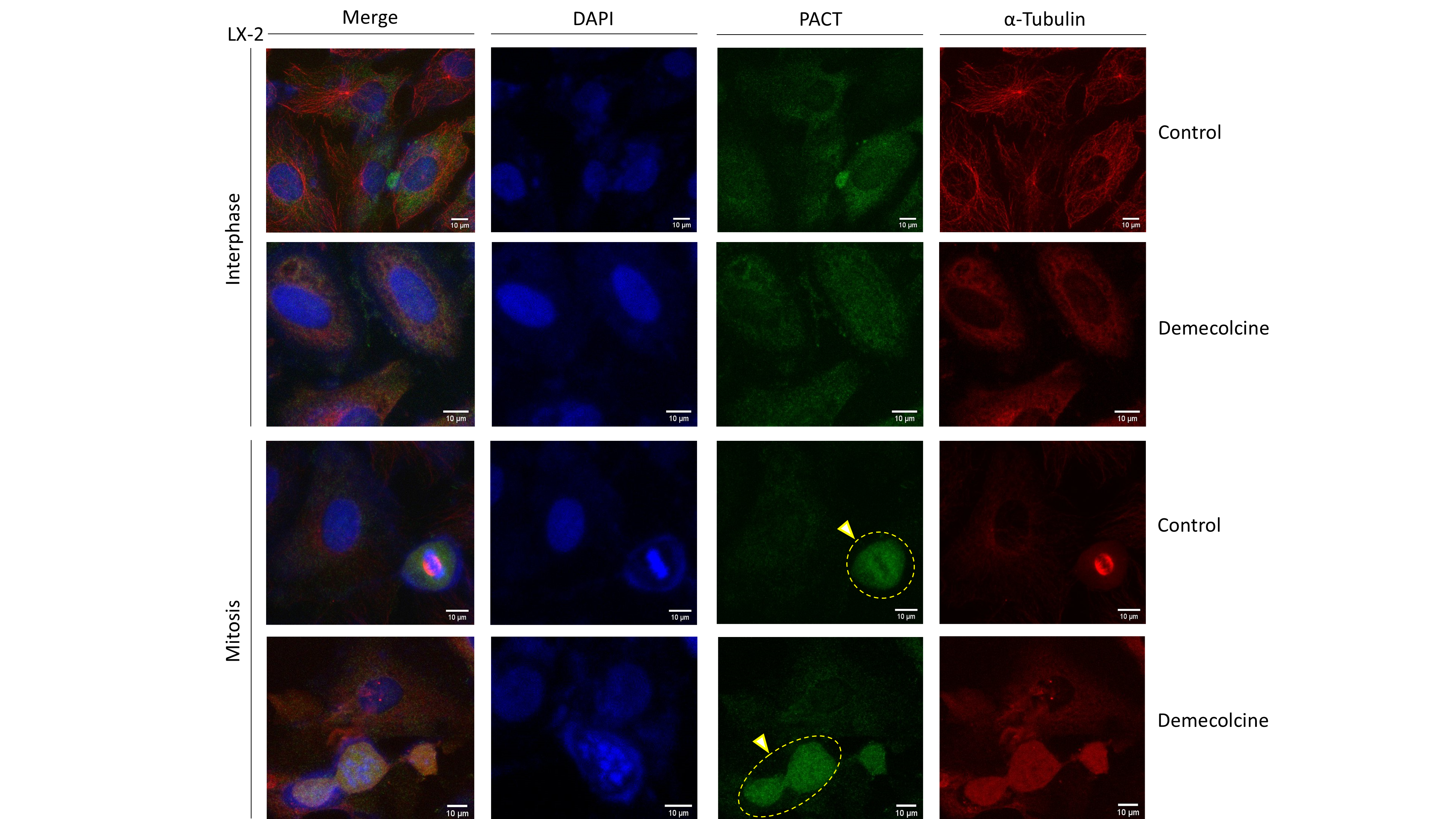

Supplement: Supplementary file 1 [file cancers-16-03701-s001.zip › S7.tif]

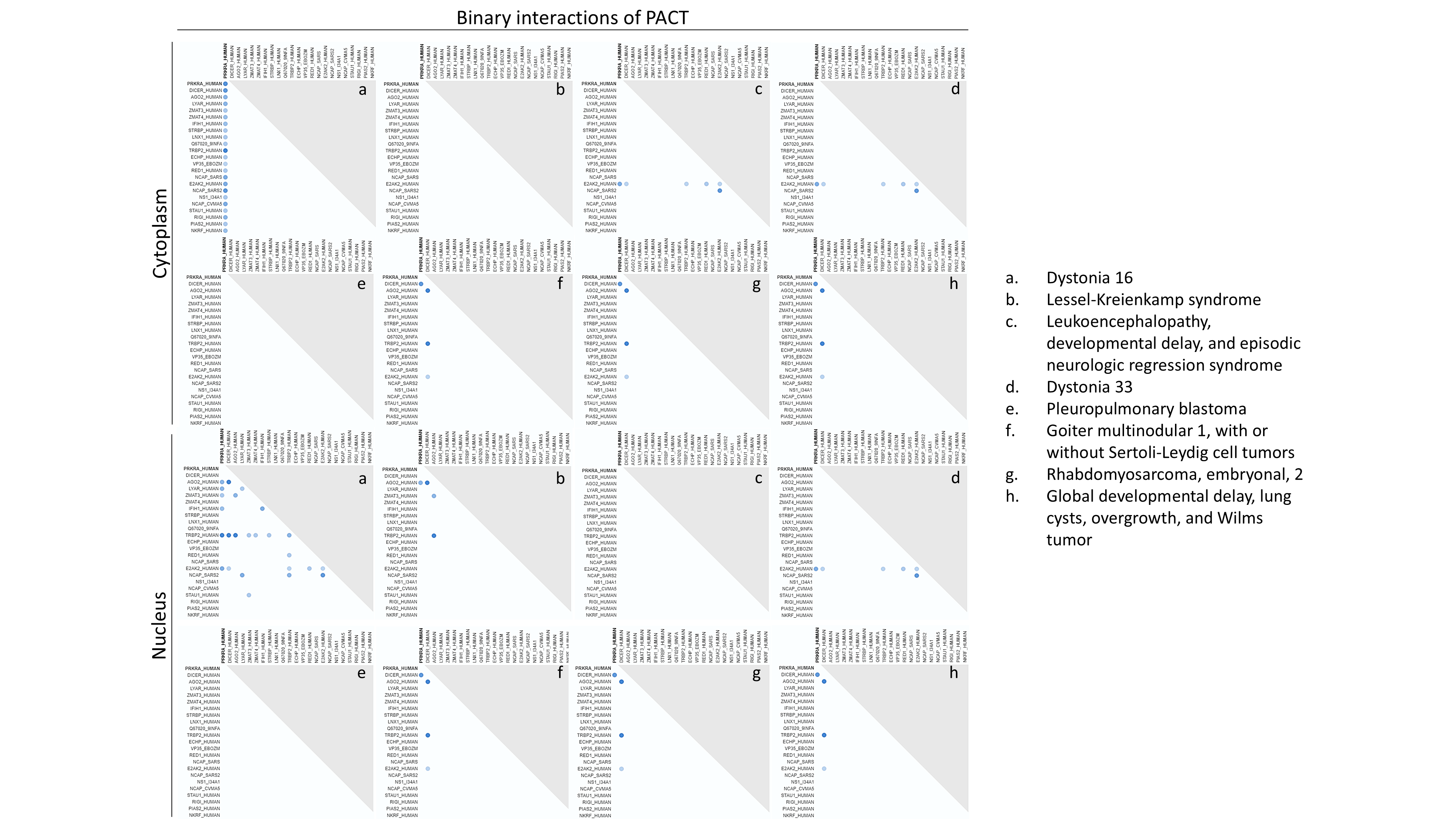

Supplement: Supplementary file 1 [file cancers-16-03701-s001.zip › S8.tif]

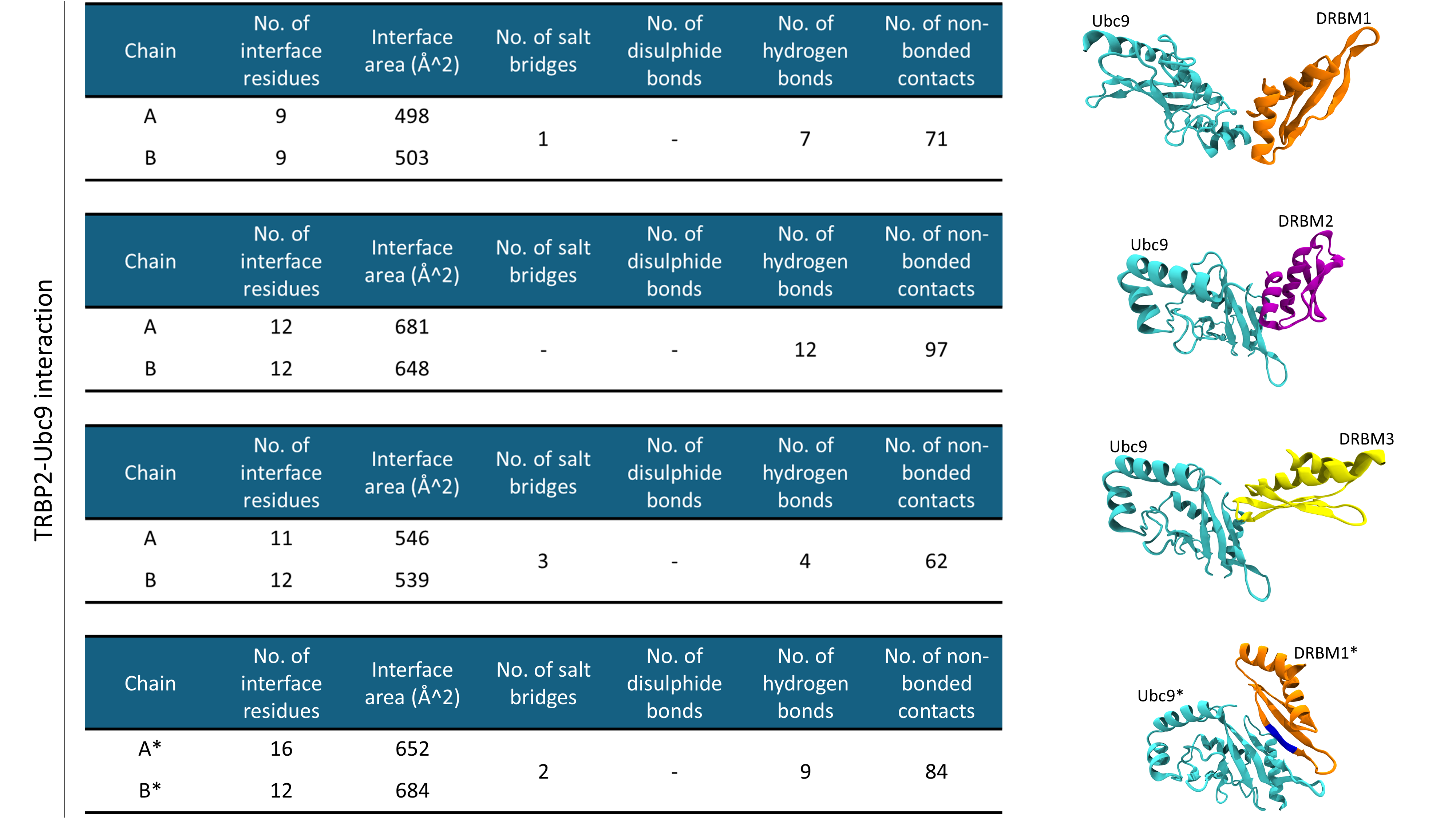

Supplement: Supplementary file 1 [file cancers-16-03701-s001.zip › S9.tif]
